# Flexible circuit-free system via passive modulated ultrasound for wireless thoracic pressure monitoring

**DOI:** 10.1126/sciadv.ads5634

**Published:** 2025-02-19

**Authors:** Muyao Wang, Lu Jia, Xinyuan Jia, Haicheng Li, Xue Feng

**Affiliations:** ^1^ Laboratory of Flexible Electronics Technology, Tsinghua University, Beijing 100084, China.; ^2^ AML, Department of Engineering Mechanics, Tsinghua University, Beijing 100084, China.

## Abstract

Implantable medical devices (IMDs) provide effective medical solutions for diverse health care applications. Electrical circuits are crucial for implantable devices due to the requirement of intended functions, such as communication with external devices. Circuits have several risks, such as biocompatibility issues, power limitations, or size constraints. In this work, we propose a passive modulated ultrasound (PMU) principle for IMDs and develop a circuit-free ultrasonic system (CUS) for thoracic pressure monitoring. The PMU principle can passively modulate monitored physiological signals into ultrasound pulses without using electrical circuits or power supply. The size of the developed CUS is only 2.5 millimeters in radius and 850 micrometers in height. Animal experiments demonstrated that the CUS, with a high sensitivity (−22.96 millivolts per kilopascal), can monitor thoracic pressure to assist in diagnosing different heart diseases, including cardiac arrest and myocardial infarction. The PMU provides a human-friendly wireless sensing and communication strategy for IMDs, which promotes advancements in health care applications within the human body.

## Teaser

Passive modulated ultrasound enables physiological monitoring without integrating circuits and batteries in implantable devices.

## INTRODUCTION

Implantable medical devices (IMDs), designed to be surgically placed into the human body, are typically used for various medical applications (*1*–*5*). Invasive surgical procedures are required to place IMDs at specific positions, and then it will exist for a period inside the human body for diagnosis, therapy, and monitoring (*6*, *7*). Implantable devices often incorporate advanced technologies such as sensors to monitor physiological parameters, electrical circuits for wireless communication and data processing, and power sources like batteries (*8*–*10*). The existing integrated circuits and batteries in IMDs pose challenges for clinical applications. The circuit and power sources dominate the volume (more than 70%) of the implantable system, resulting in a large system size and causing rejection and infection during surgery (*9*, *11*, *12*). Meanwhile, batteries and some chips contain toxic chemicals, increasing risks to biological safety and biocompatibility (*13*–*15*).

An optimal IMD, with physiological sensing and wireless communication function, is expected to be circuit-free and battery-free. A passive IMD, without extra circuits or power suppliers, can substantially reduce its biological risks and improve its work efficiency inside the body (*5*, *16*). This design can achieve a compact and small structure due to the removal of excess electronic units. Also, large penetration depth and good biocompatibility are required for IMDs to detect physiological signals inside the body, such as those in the thoracic cavity (*17*, *18*). Ultrasound has long penetration depth and high energy strength in biological tissues, providing an efficient method for wireless transmission. The excellent biocompatibility makes it an optimal route for information and energy transmission within the human body (*18*–*20*). In current ultrasound-based IMDs, the physiological signals detected by sensors cannot be directly transmitted outside the body by the ultrasonic unit due to the lack of methods for directly modulating the signal onto ultrasound carrier waves (*21*, *22*). Additional electrical circuits are required to connect the sensor and the ultrasonic unit, which transfer physiological signals collected by the sensor into a form that can be carried by ultrasound waves (*23*, *24*). The power source becomes essential for driving the circuits, ultrasonic units, and sensors. Therefore, the processing circuits and power sources are irreplaceable. Passive ultrasonic modulation refers to altering or modulating an ultrasonic signal without actively generating or amplifying the signal. It is crucial to achieve a circuit-free and battery-free ultrasound-based system.

This paper proposes a passive modulated ultrasound (PMU) principle for ultrasound-based IMDs and a circuit-free ultrasonic system (CUS) for thoracic pressure monitoring. The PMU principle uses a passive communication strategy based on the modulation of ultrasound, which can modulate the monitored physiological signals directly onto ultrasound pulses in a passive working mode. Ultrasound is used as the carrier wave for wireless sensing and communication, which experiences less attenuation in biological tissues and enhances the energy transmission efficiency within the body. The CUS, on the basis of the PMU principle, can achieve signal sensing and wireless communication simultaneously without using any electrical circuits or power supply. The absence of an internal power supply eliminates secondary surgical procedures for replacing the depleted battery. The size of the CUS is notably reduced due to its circuit-free and battery-free design, resulting in a size of only 2.5 mm in radius and 850 μm in height. Thus, the CUS substantially reduces the risk of secondary trauma and enhances its safety and biocompatibility. Animal experiments were conducted to monitor thoracic pressure during several conditions of typical heart disease, including cardiac arrest and myocardial infarction. The results showed the CUS with a strong linearity (*R*^2^ = 0.9746) and high sensitivity (−22.96 mV/kPa). This study provides prospects for ultrasound-based IMDs and has significant potential for future clinical application.

## RESULTS

### PMU principle and CUS

**Fig. 1.**
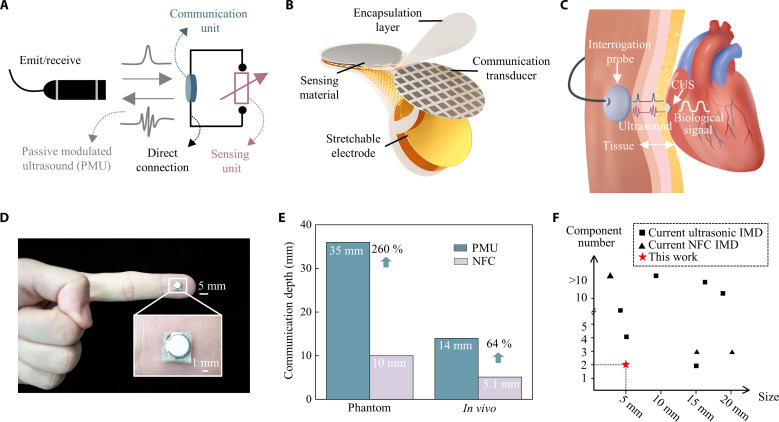
Schematic of the PMU principle and CUS. (**A**) Working process of PMU and the system composition of the CUS. The working process of PMU includes emission, modulation, and ultrasound reception. The CUS consists of only a communication unit and a sensing unit, which are connected directly. (**B**) Explode-view schematic of the CUS, including a piezoelectric transducer, a sensing material layer, a stretchable electrode layer, and an encapsulation layer. (**C**) Schematic of monitoring physiological pressure signals inside the humatn body via the CUS. (**D**) Optical diagram of the CUS, with size comparison to a human index finger. Zoom-in: system in detail. (**E**) Comparison of communication depth between the PMU and NFC methods in phantom and in vivo experiments. (**F**) Comparison of the size and component number of current IMDs.

The working process of the PMU principle includes emission, modulation, and ultrasound reception. External devices carry out the emission and reception of ultrasound, whereas the modulation of the ultrasound is performed by the CUS implanted inside the body. During modulation, the CUS responds to the emitted pulse by reflecting a modulated ultrasound pulse back to the external device based on the PMU principle. The CUS directly uses the reflected ultrasound pulse as its carrier, eliminating the need for control circuits to actively emit a dedicated ultrasound carrier. In addition, the CUS modulates the reflected ultrasound pulse via the electromechanical coupling effect, avoiding the need for control circuits to encode the ultrasound carrier. Throughout the process of PMU communication, the ultrasound carrier is not actively modulated. Because of the inherent passive characteristics of the PMU principle, the CUS consists only of a sensor unit and a communication unit, which are directly connected without using any circuit unit (Fig. 1A).

The structure of the CUS only includes a piezoelectric transducer as the communication unit and a resistance-based sensor as the sensing unit. The piezoelectric transducer and sensor were connected by a stretchable electrode and encapsulated within a flexible organic material layer. In this work, we used 1-3 piezoelectric composite materials to fabricate transducers due to their high piezoelectric coefficient (*25*). The resistance-based pressure sensor functioned as a modulator for in vivo pressure monitoring. A stretchable electrode layer and an Ecoflex substrate make the CUS flexible and stretchable. The stretchable electrode layer adopts a parallel serpentine structure, which achieves excellent mechanical flexibility and good electrical conductivity simultaneously (fig. S1). All components were encapsulated in polydimethylsiloxane (PDMS), which is soft, stretchable, and biocompatible (fig. S2) (*26*). The physiological signals within deep human tissue can be wirelessly monitored based on the PMU principle (Fig. 1C). The PMU system can be comfortably implanted at an in vivo location for sensing and communication, whereas an interrogation probe (IP) is pasted on the skin to facilitate signal exchange across the human body. The circuit-free design makes the CUS suitable for thoracic pressure due to its biocompatibility and in situ monitoring. Respiratory inductive plethysmography can monitor chest volume but cannot obtain pressure information directly (*27*). Esophageal pressure measurement can be used for assessing thoracic pressure, but this method requires an invasive insertion procedure and cannot measure thoracic pressure in situ (*28*). Thus, the CUS has advantages in energy efficiency and biocompatibility for thoracic pressure monitoring.

The system can be folded along the center by 180°, causing the sensors and transducers to be tightly contacted back-to-back, thereby reducing the system’s overall size (Fig. 1D). Also, by eliminating the need for electrical circuits and batteries, the CUS reduces its size to only 2.5 mm in radius. Near-field communication (NFC) is a wireless communication method based on electromagnetic waves, but its transmission efficiency is greatly limited in biological tissues. The absorption and attenuation of electromagnetic waves are severe in biological tissues (−3 dB/cm) (*29*), resulting in an NFC distance typically being less than 5 mm (*30*, *31*). Ultrasound as a mechanical wave has less attenuation in biological tissues (−1 dB/cm) (*32*) and has better penetration ability inside the body (see more details in note S1 and fig. S3). The PMU principle based on ultrasound has a deeper transmission distance and better biocompatibility than NFC within the human body. The PMU system achieved a communication depth of up to 35 and 14 mm in phantom (water) and living organisms, respectively, which is 250 and 64% higher than that of NFC, respectively (Fig. 1E). The PMU principle allows the CUS to monitor physiological signals without using both the energy storage and microcontroller unit (MCU) inside the body. A detailed comparison between current IMDs and PMU devices is illustrated in note S2 and fig. S4. Because of the inherent passive characteristics, the CUS is notably simpler and smaller compared to most current implantable biomedical systems (IBSs) (Fig. 1F) (*12*, *19*, *21*–*23*, *29*, *33*–*35*).

### Mechanism of the PMU principle

The mechanism of PMU provides a theoretical basis for implantable circuit-free systems to achieve monitoring functions. The PMU mechanism reconstructs monitored physiological signals by analyzing the characteristics of reflected ultrasound pulses.

**Fig. 2.**
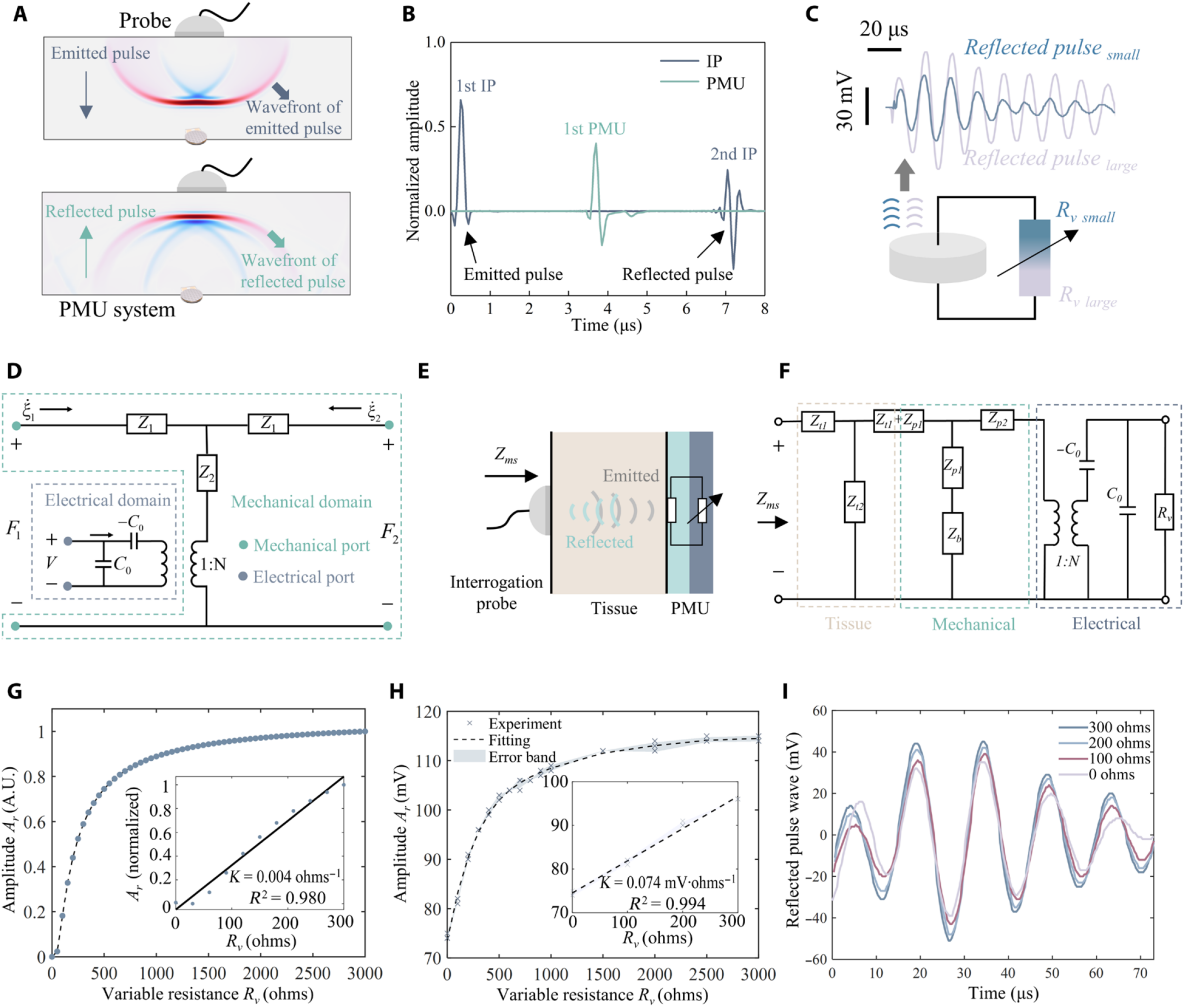
Mechanism of the PMU principle. (**A**) Acoustic field simulation and (**B**) the ultrasound pulse sequence in PMU. (**C**) Amplitude modulation principle of the piezoelectric transducer. (**D**) Three-port network model for the piezoelectric transducer in the PMU system. (**E**) Schematic diagram and (**F**) network model of the PMU system for signal monitoring in deep human tissue. (**G**) Theoretical and (**H**) experimental results of amplitude modulation law based on the network model. (**I**) Time-domain waveforms of reflected ultrasound pulses after amplitude modulation. A.U., normalized variable.

The acoustic field transmission in one PMU period is simulated, and visualization of the ultrasound propagation is shown in Fig. 2A. The IP emits an ultrasound pulse, which transmits through human tissue toward the PMU system. The PMU system’s reflected ultrasound pulse will be returned to physiological information. The ultrasound pulse sequence is shown in Fig. 2B, and blue and green lines represent ultrasound pulses of the IP and the PMU system, respectively. The first IP and first PMU are the ultrasound pulses emitted by the IP and received by the PMU system, respectively. The second IP is the reflected pulse the IP receives, which carries monitored physiological information.

The amplitude modulation for communication is based on the electromechanical coupling effect of piezoelectric material (Fig. 2C) (*36*, *37*). The energy of the emitted ultrasound pulse received by the PMU system is transferred into two modes (fig. S5). One portion of the energy is reflected backward, forming the reflected ultrasound pulse (ultrasonic mode); the other portion of the energy is converted into electrical energy through the piezoelectric effect, which is then depleted on the external electronic components connected with the transducer (electrical mode).

Traditional ultrasonic implantable systems focus on maximizing the energy of electrical mode by adjusting the external impedance to an optimal condition (*13*, *38*). However, for a PMU system, electrical energy dissipation on the external electronic components (electrical mode) should be adjusted to change the energy of reflected ultrasound pulses (ultrasonic mode). The energy of the electrical mode can be altered by adjusting the impedance of external electronic components. Meanwhile, the electrical and ultrasonic energy modes exhibit a negative correlation when the input energy is constant. For the PMU system, the external impedance is a variable resistor *R_v_*. Thus, the electrical energy dissipation (electrical mode) is more substantial with a smaller *R_v_*, leading to a smaller amplitude of reflected ultrasound pulses (ultrasonic mode). Therefore, the amplitude of the reflected ultrasound pulse can be changed by changing *R_v_*, which is the amplitude modulation of PMU.

A network model is established to describe the PMU principle theoretically. The piezoelectric transducer in PMU can be described as a three-port network based on the piezoelectric equation and vibration equation (Fig. 2D). The detailed theoretical solution of the network model is shown in note S3. This three-port network can be divided into a mechanical and an electrical domain connected by an electromechanical converter (1:N). The mechanical and electrical domains of the network are associated with the energy of ultrasonic mode and electrical mode, respectively. The PMU system consists of a piezoelectric transducer and a variable resistor *R_v_* connected in series. This indicates that the network model of the PMU system is a variable resistor *R_v_* connected in series with the transducer network at the electrical port (fig. S6A). The schematic of the monitoring system based on PMU inside the human body is shown in Fig. 2E, which consists of an IP, a tissue layer, and the PMU system. The tissue layer is described as a network with only a mechanical domain, as shown in fig. S6B. Therefore, the network model of the monitoring system is established by cascading the network of the tissue layer and the PMU system, as shown in Fig. 2F. Impedance of the monitoring system (*Z_ms_*) can be derived from Fig. 2F as

$$Z_{\mathrm{ms}}=\frac{Z_{t0}\left[Z_{PMU}+jZ_{t0}\text{tan}(\frac{kl_{t}}{2})\right]}{j\text{sin}\left({\mathrm{kl}}_{t}\right)\left[Z_{PMU}+jZ_{t0}\text{tan}\left(\frac{kl_{t}}{2}\right)+\frac{Z_{t0}}{j\text{sin}\left(kl_{t}\right)}\right]}$$ (1)

where *Z*_*t*0_ is the acoustic impedance of the tissue layer, and *Z_PMU_* is the impedance of the PMU system network. *L_t_* is the tissue layer thickness, and *k* is the wave number. The amplitudes of reflected ultrasound pulses (*A_r_*) corresponding to different *R_v_* can be derived based on Eq. 1 (see more details in note S4). The theoretical relationship between *A_r_* and *R_v_* is shown in Fig. 2G. There is a linear relationship (*R*^2^ = 0.980) between *A_r_* and *R_v_* within the 0- to 300-ohm range. The PMU system uses this linear region (0 to 300 ohms) to achieve communication. In vitro experiment was conducted to demonstrate the PMU principle, and the setup is shown in fig. S7. The experimental relationship between *A_r_* and *R_v_* is shown in Fig. 2H. There is also a linear relationship (*R*^2^ = 0.994) between *A_r_* and *R_v_* within the 0- to 300-ohm range. Experimental results are consistent with theoretical results, proving the feasibility and correctness of the PMU principle. Compared to theoretical results in Fig. 2G, experimental results in Fig. 2H show that *A_r_* is a finite value rather than zero when *R_v_* is 0 ohms. This discrepancy is attributed to the presence of the substrate interface in the experimental setup, which does not exist in the theoretical model (see more details in note S5 and fig. S8). On the basis of this phenomenon, we can distinguish between the reflection from the substrate and the PMU system (see more details in note S5 and fig. S8). Figure 2I shows experimental results of time-domain waveforms of reflected ultrasound pulses. The *A_r_* of waveforms under different *R_v_* values is considerably different. Spectral analysis was conducted on these waveforms. Results indicate that the reflected pulse amplitude (*A_r_*) increases with the increase in resistance *R_v_* (fig. S9A). The resonant frequency shows no significant variation with the increase in resistance *R_v_* (fig. S9B). Thus, the amplitude of the reflected ultrasound pulse is used as the modulation parameter for the PMU principle. The transmission distance can affect the reflected pulse amplitude, such as the tissue thickness of patients. The reflected pulse amplitude can be corrected based on the attenuation law of ultrasound, which eliminates the specific influence of transmission distance (see more details in note S6). Meanwhile, it was observed that the spectrum of reflected pulse with *R_v_* = 0 ohms displayed two peak frequencies, which is induced by the superposition of CUS surface reflection and interface reflection (see more details in note S7 and fig. S9, B and C).

### Design and performance of CUSs

**Fig. 3.**
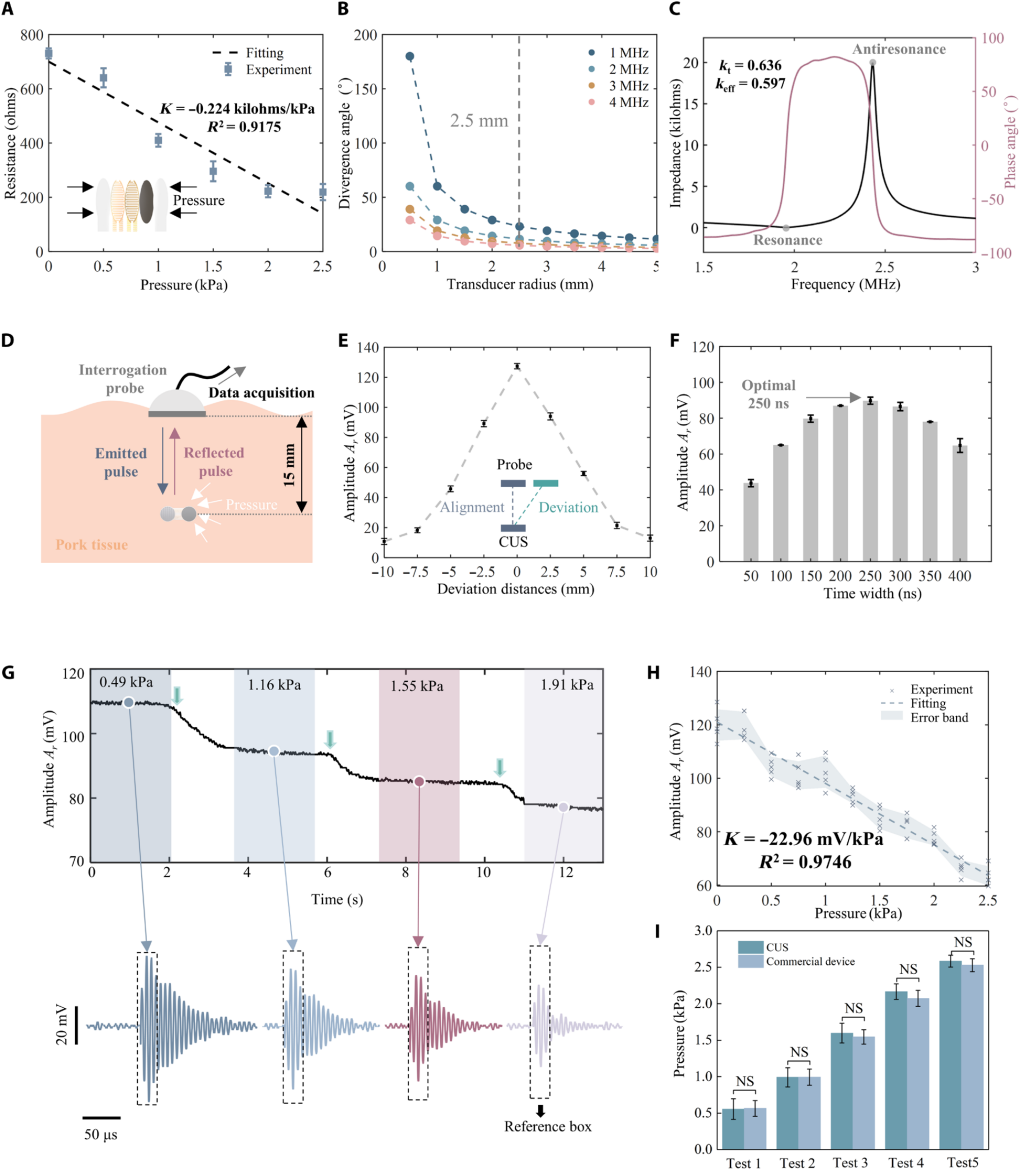
Design and performance of the CUS. (**A**) Calibration curve of the piezoresistive pressure sensor, with an inset showing the sensor structure. (**B**) Impedance analysis results of the CUS’ transducer. (**C**) Trade-off between the frequency and size of the transducer indicating that a 2-MHz frequency and a 2.5-mm radius are the optimal solutions for the CUS. (**D**) Schematic diagram of in vitro experiment setup for CUS characterization. (**E**) Reflected pulse amplitudes at different lateral deviation distances under a fixed emitted pulse amplitude. (**F**) Time-domain optimization of the CUS, indicating that a pulse width of 250 ns leads to the strongest response level. (**G**) Reflected pulse amplitude varying with the increasing loading pressure (upper), and the reflected pulse waveforms at different pressures (lower). The black dashed box is used to compare these four waveforms. (**H**) Calibration curve of the CUS for pressure monitoring in deep tissue. (**I**) Comparison of pressures measured by the CUS and a commercial sensor. NS, not significant.

In this work, CUSs were designed based on the PMU principle and used to monitor the organ pressure in deep tissue due to its importance in the clinic. Here, we used a resistance-based pressure sensor as the variable resistor *R_v_* in PMU. When pressure is loaded onto the sensor, the conductivity of the sensitive layer increases (*39*), decreasing the sensor’s resistance (Fig. 3A).

Both the frequency and size of the transducer in the CUS affect its communication performance. The divergence angle (DA) of the transducer’s main lobe is reduced to optimize transmission efficiency. The theoretical analysis of the DA of the main lobe is introduced in note S8. The DA has a negative correlation with the frequency under fixed transducer size conditions, as shown in Fig. 3B. However, the energy intensity of ultrasound decreases proportionally with increasing frequency in human tissues, which means the working frequency of the transducer cannot be too high. The transducer frequency is chosen as 2 MHz, which is the optimal solution to achieve a small DA and high energy intensity simultaneously. The size of the transducer is set as a 2.5-mm radius, which maintains a small system size and avoids substantial expansion of DAs under each frequency (note S9).

The CUS adopted 1-3 piezoelectric composite material to fabricate the transducer due to the high piezoelectric coefficient (*d*_33_ ≈ 350 × 10^−12^ C/N) and good acoustic impedance matching (15.3 megarayleighs) (fig. S10) (*38*). The composite was processed into a circular shape as the CUS transducer, which has a better acoustic field directivity than that of a square shape transducer (fig. S11). The impedance analysis (Fig. 3C) indicates the resonant (*f_r_*) and antiresonant (*f_a_*) frequencies were 1.95 and 2.43 MHz, respectively. The transducer has an electromechanical coupling coefficient *k_t_* of 0.636 and an equivalent coupling coefficient *k_eff_* of 0.597.

An in vitro experiment system demonstrated the pressure monitoring performance of the CUS (Fig. 3D). The lateral deviation between the IP and CUS can reduce the reflected pulse amplitude due to the acoustic field directivity of the IP. Figure 3E illustrates reflected pulse amplitudes at different lateral deviation distances under a fixed emitted pulse amplitude. Experimental results indicate that, when the CUS is aligned with the probe, the reflected pulse amplitude is maximized, representing the optimal value. The amplitude reduction is within 30% when the lateral deviation distance is within 5 mm. The tilt angle of the CUS can also affect the reflected pulse amplitude. Figure S13 shows reflected pulse amplitudes at different tilt angles under a fixed emitted pulse amplitude. Experimental results revealed that the decrease in amplitude is less than 6.5 and 7.3% when the tilt angle of the CUS is within ±5°, indicating a relatively minor impact. Experiments were further conducted to measure reflected pulse amplitudes with different bending conditions under a fixed emitted pulse amplitude (fig. S14). There is no significant difference in measurement results under bending deformation of 0°, 90°, and 180°, demonstrating the CUS’ stable pressure monitoring performance under bending conditions.

The CUS is an equivalent mechanical impedance system, and the reflected pulse amplitude *A_r_* is affected by the time width of the emitted pulse (fig. S15). Figure 3F shows *A_r_* corresponding to different time widths of the emitted pulse. The optimal pulse time width is 250 ns for achieving the highest reflected pulse amplitude under each *R_v_* value. Therefore, the time width of the emitted pulse was set as 250 ns to achieve optimal communication sensitivity. Results also indicate that the CUS has different responses under different time widths of the emitted pulse. This property can be further used to encrypt information obtained from CUS and enhance the security of PMU communication.

A customized IP (UT2C4, Doppler, China) was used to communicate with the CUS, which emits and receives reflected ultrasound pulses from the CUS. The interrogation probe is designed to operate at 2 MHz with a diameter of 6.5 mm. The IP has a high sensitivity (*K* = 13.20 kPa/V), good linearity (*R*^2^ = 0.99), and a wide bandwidth (~43.92%), enabling it to capture amplitude variation of the reflected pulse (fig. S16, A and B). The sound field generated by the IP has high intensity and uniform distribution (fig. S16, C and D). Meanwhile, the noise of the IP is very minimal, ensuring the accuracy of received ultrasound pulse signals (fig. S17).

Figure 3G (top) shows the pressure signal monitored continuously in vitro by the CUS, which was reconstructed from the reflected ultrasound pulses based on PMU. The pressure was gradually loaded to the CUS, with values of 0.49, 1.16, 1.55, and 1.91 kPa. The reflected pulse waveforms of these four stages received by the IP are shown in Fig. 3G (bottom). The amplitudes of the reflected pulses in the four stages show notable differences, with 109.43, 94.27, 85.10, and 77.09 mV at pressures of 0.49, 1.16, 1.55, and 1.91 kPa, respectively. The calibration curve of the CUS shows good linearity (*R*^2^ = 0.9746), high sensitivity (*K* = −22.96 mV/kPa), and low nonlinearity (δ = 9.53%) (Fig. 3H). The accuracy of the CUS has demonstrated in contrast with a commercial pressure monitoring device (FSR-4 channel, WAAX, China). No significant difference is observed between the measurement results of the CUS and the commercial device, demonstrating the accuracy and stability of the CUS (Fig. 3I).

To investigate the dynamic response performance of the CUS, high pressure (1 kPa) and low pressure (0.5 kPa) were applied to the CUS for loading and unloading tests with three cycles performed (fig. S18A). In addition, pressures (1 kPa) with different frequencies of 1 and 3 Hz were applied to the CUS (fig. S18B). We further demonstrated the ability of the CUS in encoding ultrasonic communication based on the ASCII encoding protocol (fig. S19). The impact of the folding state on CUS monitoring was also investigated. Experiments were conducted to verify the pressure monitoring performance of the CUS in both folded and unfolded states (fig. S20, A and B). Results indicated that the waveform results of the CUS in both folded and unfolded states are similar under the same pressure (fig. S20, C and D). Also, there was no significant difference in pressure monitoring performance between the folded and unfolded states of the CUS (fig. S20E).

### Thoracic pressure monitoring

**Fig. 4.**
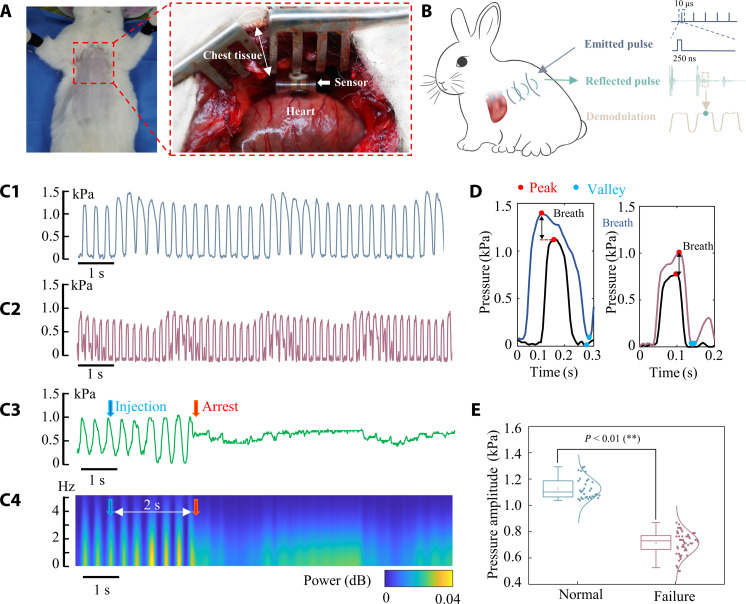
In vivo experiment for thoracic pressure. (**A**) In vivo experiment setup of thoracic pressure monitoring for heart failure. (**B**) Data processing flow for thoracic pressure monitoring through PMU. [(**C**), 1] to [(C), 2] Rabbit’s normal thoracic pressure, thoracic pressure after injecting dopamine to induce heart failure, and thoracic pressure when injecting KCl solution to induce cardiac arrest, respectively. [(C), 4] Spectral analysis of [(C), 3], showing changes in thoracic pressure during the cardiac arrest period. (**D**) One period of thoracic pressure in the normal state (left) and heart failure state (right). (**E**) Statistical analysis of thoracic pressure amplitudes in normal and heart failure states, showing a significant decrease in amplitude under the heart failure condition.

Thoracic pressure is an important physiological health indicator, especially crucial for assessing the health status of the cardiovascular system (*40*). The CUS is able to monitor thoracic pressure signals, which can provide early warning and diagnosis of diseases such as cardiac arrest and myocardial infarction by revealing abnormal thoracic pressures. We designed an animal experiment to validate the CUS’ effectiveness in monitoring thoracic pressure. The CUS was surgically implanted inside the subcutaneous tissue of a rabbit’s chest. Initially, the CUS sensor was securely affixed onto the rabbit’s heart surface using surgical dressings, and thoracic pressure and electrocardiogram (ECG) were recorded simultaneously. Subsequently, the CUS was securely affixed to the rabbit’s heart outer surface using surgical dressings. Following the implantation of these devices, the opened chest was carefully sutured. Detailed illustrations depicting the device placement in the in vivo experiment are provided in Fig. 4A. The thoracic pressure signal is monitored by the CUS and wirelessly transmitted outside through ultrasound. Then, the monitored pressure signal can be reconstructed through demodulation of these reflected ultrasound pulses (Fig. 4B).

Both thoracic pressure and heart rate maintained normal cyclic variations under conditions of no interference to the heart, as shown in Fig. 4C1. The thoracic pressure ranged from 0 to 1.49 kPa, with a corresponding heart rate of 3.36 Hz. The waveform exhibited fluctuations of 0.25 Hz, reflective of periodic changes in thoracic pressure attributed to respiration. The tested curve of the ECG signal is provided in fig. S21. The heart rates obtained from PMU correspond to those from the ECG signals, validating the accuracy of the CUS.

Then, dopamine (20 mg diluted in 500 ml of physiological saline) was injected into the ear veins of the rabbits to cause heart failure. An IP was pasted onto the skin of the chest for communicating with the CUS. The thoracic pressure signal during the state of heart failure is presented in Fig. 4C2. Thoracic pressure ranged from 0 to 1.044 kPa during heart failure, whereas the heart rate increased to 5.82 Hz. In addition, the waveform exhibited increased fluctuations, with respiration causing fluctuations of 0.39 Hz. The heart rates from ECG and our PMU are still the same, demonstrating the CUS’ accuracy. These results demonstrate that the CUS can detect accurate pressure in normal and abnormal heart status.

Last, KCl solution (10 ml) was injected through the ear vein of the rabbit, leading to cardiac arrest. The thoracic pressure remained within 0 to 1 kPa before the cardiac arrest. An obvious decrease in pressure occurred after ~2 s of injection, and the cyclic variation in pressure disappeared (Fig. 4, C3 and C4). Despite the occurrence of cardiac arrest, the thoracic pressure remained around 0.6 kPa instead of 0 kPa. This phenomenon is due to hyperkalemia caused by the KCl injection, which disrupted the electrical potential of myocardial cells (*35*). Also, myocardial cells cannot contract normally and lead to cardiac arrest during the diastolic state (*40*–*42*).

One period of thoracic pressure in normal (left) state and heart failure (right) state are shown in Fig. 4D. Results indicate that the inhale stage can improve the pressure amplitude. Statistical analysis results demonstrate that the thoracic pressure amplitude reveals a significant decrease in heart failure than usual (Fig. 4E).

**Fig. 5.**
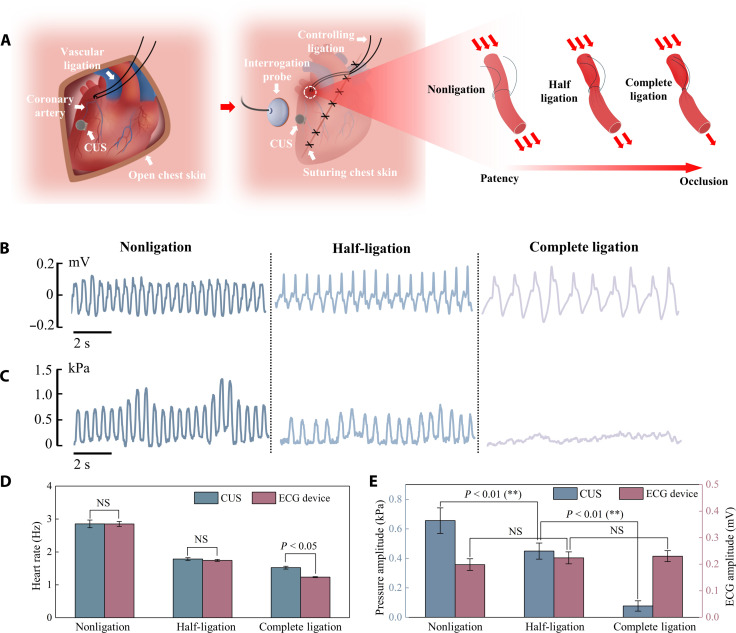
In vivo experiment for monitoring myocardial infarction. (**A**) Experimental setup of thoracic pressure monitoring of myocardial infarction. Zoom-in: Schematic of vessel states with nonligation, half-ligation, and complete ligation. (**B**) Rabbit’s ECG signal and (**C**) thoracic pressure during the nonligation, half-ligation, and complete ligation stages. (**D**) Comparison of heartbeat frequency between results measured by the CUS and commercial ECG device. (**E**) Comparison of early prediction ability for myocardial infarction by the CUS and commercial ECG device.

Myocardial infarction, commonly known as a heart attack, occurs when the blood flow at a part of the heart muscle (myocardium) is blocked for a prolonged period, which can cause permanent damage to the heart (*40*, *43*). The CUS can provide early warning and diagnosis of myocardial infarction by monitoring the thoracic pressure in real time. A myocardial infarction experiment was designed to demonstrate the clinical application of the CUS (Fig. 5A). Myocardial infarction was caused by coronary artery ligation, which reduces the diameter of the coronary artery and causes insufficient blood supply to the heart. Then, the severity of myocardial infarction was controlled by the coronary artery diameter after ligation. The suture wrapped around the vessel for one loop, forming a ligation on the vessel. The suture can cause vessel occlusion, thus simulating different states of myocardial infarction. The ECG signal in nonligation, half-ligation, and complete ligation is shown in Fig. 5B. The frequency of the ECG signal obviously changes from 2.85 to 1.24 Hz as the blood vessels ligate entirely. This indicates that coronary artery ligation significantly affects the beating of the heart. However, the three states have no significant difference in ECG signal amplitude and failed to warn of the myocardial infarction early. The thoracic pressure in states of nonligation, half-ligation, and complete ligation is shown in Fig. 5C. As the degree of ligation increases, the pressure amplitude gradually reduces. This is attributed to insufficient blood supply weakening the heart’s beating ability (*44*). In terms of frequency, the thoracic pressure signal matches the ECG signal. The pressure trend is consistent with the trend of breathing, which is not detected by the ECG signal. As the diameter of the coronary artery decreases, the frequency of the thoracic pressure signal also significantly decreases from 2.85 to 1.52 Hz. Results also demonstrate a significant influence of coronary artery ligation on heartbeating. Meanwhile, the amplitude of thoracic pressure also decreases significantly, showing the ability to warn early for myocardial infarction.

The comparison of heartbeat frequency measured by the CUS and a commercial ECG device is shown in Fig. 5D. In the three stages, the heart rate measured by both devices showed a significant downward trend. In stages of nonligation and half-ligation, there was no significant difference in results between the CUS and ECG devices (double-tailed *t*-distribution statistics). In the stage of complete ligation, there is a difference in the frequency measured by the CUS due to the thoracic pressure becoming disordered in the complete ligation state. The comparison of ECG signal amplitude and thoracic pressure amplitude is shown in Fig. 5E. The amplitude of the ECG signal shows no significant difference in the three states. Thus, the ECG cannot be used for diagnosis and early warning of myocardial infarction. However, there are substantial differences in the amplitude of thoracic pressure monitored by the CUS, which can diagnose the degree of myocardial infarction and provide timely warning for the occurrence of myocardial infarction.

## DISCUSSION

We have proposed the principle of PMU and developed CUSs for thoracic pressure monitoring. The mechanism of PMU is illustrated based on the electromechanical coupling effect, and a theoretical model is proposed to facilitate CUS design. The developed CUS adopted only two passive components to achieve sophisticated monitoring functionality. The PMU principle enabled the CUS to have a compact size of only 2.5 mm in radius and 850 μm in height. Animal experiment results demonstrated the CUS’ capabilities of monitoring and diagnosing several heart disease conditions, including cardiac arrest and myocardial infarction. This research could be a guide for IMDs to achieve passive sensing and communication, and it could open up broad prospects for enhancing the clinical performance of IBSs.

## MATERIALS AND METHODS

### Fabrication of CUSs

The manufacturing process for the CUS includes three main steps: initially fabricating the stretchable electrode layer, integrating components onto it, and lastly encapsulating the assembly. In the initial step, a polymethyl methacrylate (PMMA) film serving as a sacrificial layer was spun onto a silicon wafer and cured. Subsequently, a polyimide (PI) layer is spined onto PMMA and cured. Then, electron beam evaporation deposited a metal layer consisting of 20-nm Cr and 200-nm Au onto the PI film. The metal electrode was etched using lithography principles. Then, the Au/Cr-PI-PMMA layer was peeled off from the silicon wafer and transferred onto a PDMS film. Components of the CUS included a piezoelectric transducer and a resistance-based pressure sensor. A circular-shaped 1-3 composite piezoelectric material was fabricated as the transducer. In the second step, the transducer was affixed to the stretchable electrode by conductive silver paste. Then, the PI film was etched in the same pattern as the stretchable electrode via reactive ion etching. The pressure sensor used a layer of graphene as the sensitive material layer, converting pressure signals into resistance signals (XFNANO Materials Tech, China). In the final step, the assembly comprising the stretchable electrode layer and components was encased within a mold filled with fluid PDMS for encapsulation (70°C for 1 hour). After that, the CUS was fabricated, as shown in Fig. 1D.

### Simulations of acoustic fields

The acoustic field simulation was conducted using multiphysics finite element analysis (FEA) software (Comsol 6.0). In the simulation of the PMU process (Fig. 2A), the vertical spacing between the interrogating probe and the CUS was 20 mm. The transmission medium was water, using linear elasticity. The temperature was set at 293.15 K. Ultrasonic transducers of the IP and CUSs were simplified as plane wave sound sources.

### Electromechanical characterization of the piezoelectric transducer

The impedance and phase angle curves were measured via a commercial impedance analyzer (ZX70A, New Precision Electronics Co., China) with a scanning range of 10 kHz to 10 MHz. The resonant frequency *f_r_* and antiresonant frequency *f_a_* correspond to the minimum and maximum values on the impedance curve, respectively (Fig. 3C). The *k_t_* represents the efficiency of energy conversion between electrical and mechanical forms in piezoelectric materials. The *k_eff_* quantitatively assesses the electromechanical efficiency of transducers. The *k_t_* and *k_eff_* were derived from Eqs. 2 and 3, respectively (*45*)

$$k_{t}=\sqrt{\frac{\pi }{2}\frac{f_{r}}{f_{a}}\text{tan}\left(\frac{\pi }{2}\frac{f_{a}-f_{r}}{f_{a}}\right)}$$ (2)

$$k_{\mathrm{eff}}=\sqrt{1-\frac{f_{r}^{2}}{f_{a}^{2}}}$$ (3)

### Animal experiments

All animal experiments were performed at the Beijing Medical Services Biotechnology (Beijing, China) and approved by the Ethics Committee of Beijing Medical Services Biotechnology (MDSW-2023-065C). New Zealand white rabbits were anesthetized via intraperitoneal injection of propofol (10 mg/kg) until the dosage took effect. Then, tracheal intubation was performed to connect a respiratory anesthesia machine and maintained anesthesia with a 2 to 3% concentration of isoflurane. The rabbit was positioned in the supine posture, with the chest skin shaved. Subsequently, a thoracotomy was performed to expose the heart. The ultrasonic pulse data of PMU were recorded by ultrasonic control equipment (Multiscan-4, Doppler, China). Surgical dressings were used to affix the CUS onto the outer surface of the rabbit’s heart. ECG signals of the rabbit were obtained using the four-limb lead method. Puncture needles (NE-S-1500, Friend Ship Medical, China) were inserted subcutaneously into the limb and chest for ECG monitoring. ECG signals were recorded through a data acquisition system (BL-420F, Chengdu Techman Soft, China).

### Statistical analysis

Statistical significance was assessed using a two-tailed unpaired *t* test for experiments with only two conditions (Figs. 3I, 4E, and 5D). For experiments involving three conditions, pairwise *t* tests were conducted (Fig. 5E). *P < 0.05 is considered to have statistical significance, and **P < 0.01 is considered to have significant statistical significance.

## Supplementary Materials

### This PDF file includes:

Supplementary Notes S1 to S9 Figs. S1 to S21 References
