# Supplementary material for: Flexible circuit-free system via passive modulated ultrasound for wireless thoracic pressure monitoring
Source: Sci Adv. 2025 Feb 19;11(8):eads5634. doi: 10.1126/sciadv.ads5634 (PMC11837986; doi:10.1126/sciadv.ads5634)
Supplement: Supplementary file 1 — Supplementary Notes S1 to S9 Figs. S1 to S21 References [file sciadv.ads5634_sm.pdf]

Science Advances
AAAS

Supplementary Materials for

Flexible circuit-free system via passive modulated ultrasound for wireless thoracic pressure monitoring

Muyao Wang *et al.*

Corresponding author: Haicheng Li, thulhc@tsinghua.edu.cn; Xue Feng, fengxue@tsinghua.edu.cn

Sci. Adv. **11**, eads5634 (2025)
DOI: 10.1126/sciadv.ads5634

This PDF file includes:

Notes S1 to S9
Figs. S1 to S21
References

Note.S1. Comparison of transmission performance of electromagnetic waves and ultrasound in biological tissues.

NFC is a widely used passive communication method based on electromagnetic waves (EM). The attenuation of electromagnetic waves in air is relatively weak, allowing NFC communication to reach several centimeters. However, biological tissues have strong absorption effects on EM, which severely reduces the NFC communication distance *in vivo*. The attenuation of electromagnetic wave energy with transmission distance can be described as follows:

$$A=A_0\times e^{-\beta x} \tag{S1}$$

where A_0 and A are the amplitude at the initial position and distance x, respectively. And β represents the attenuation coefficient of electromagnetic waves in a specific medium (β = -3 dB/cm for electromagnetic waves in biological tissues (*32*). The results of equation (S1) are shown in Fig.S3, where the -3dB transmission distance of electromagnetic waves is only 9.9 mm in biological tissues, corresponding to a communication distance of about 5 mm. In contrast, ultrasound has less attenuation in biological tissues (β = -1 dB/cm) (*29*), allowing a -3dB transmission distance of 29.9 mm. Current implantable devices based on NFC and similar size to CUS, generally have a communication distance of about 5 mm *in vivo* (*46-48*). Therefore, the communication depth of NFC is considered to be 5 mm *in vivo*.

Note.S2. Comparison between current implantable medical devices and the PMU device.

A typical implantable medical device includes the sensor, processing circuit, and transducer (Fig.S4a) (*49, 50*). The processing circuit consists of a battery and a microcontroller unit (MCU). The battery supplies power to the MCU, sensor, and transducer, enabling them to achieve the functions of signal sensing, signal processing, and signal transmission, respectively. The MCU is the core component for IMD to implement communication, which sequentially controls the signal sensing, signal processing, and signal transmission. It drives the sensor to acquire signals and encode information, then drives the transducer to emit modulated ultrasound outward. This system relies on a battery to supply power and an MCU to modulate signals, which adopts the active method in both power supply and communication.

Another typical implantable medical device uses a wireless power supply (electromagnetic (*51*), ultrasound (*23*), triboelectricity (*52*), etc.), which eliminates the need for built-in batteries (Fig.S4b). However, these devices still rely on MCU to encode signals and modulate them into ultrasonic carriers for communication. Therefore, these devices are passive in terms of power supply, but still active in terms of communication.

The implantable medical devices based on the PMU principle allow the sensor and transducer to be connected directly for communication, eliminating the usage of both the battery and MCU (Fig.S4c). The PMU principle utilizes reflected ultrasonic pulses as carriers and regulates the reflected wave energy through a resistor R_v to achieve amplitude modulation. The monitored physiological signals are directly modulated into the reflected pulses through the resistance R_v of the sensor. This modulation to the reflected pulse does not require the MCU to encode the signal and re-modulate the ultrasound carrier, indicating that the PMU principle is a passive modulation method. Therefore, devices based on the PMU principle are passive in terms of both power supply and communication.

Note.S3. The detailed theoretical solution of the network model.

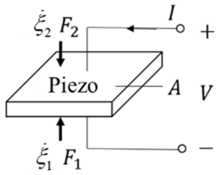

The velocity and force of the upper surface are denoted as $\dot{\xi}_2$, F_2; and the force and velocity of the lower surface are denoted as $\dot{\xi}_1$, F_1. The piezoelectric equation is as follows：

$$\begin{cases} T = cS - hD \\ E = -hS + \beta D \end{cases} \tag{S2}$$

where *T*, *S*, *D* and *E* are stress, strain, electric displacement and electric field intensity of the piezoelectric transducer, respectively. The *c*, *h* and *β* are elastic coefficient, piezoelectric coefficient and dielectric coefficient of the piezoelectric material, respectively. The vibration equation of the piezoelectrical transducer is:

$$\rho \frac{\partial^2 \xi}{\partial t^2} = \frac{\partial T}{\partial z} \tag{S3}$$

The control equation of the network model is obtained by combining equation (S2) and (S3):

$$\begin{cases} F_1 = jZ_0 \tan\frac{kl}{2}\dot{\xi}_1 + \frac{Z_0}{j\sin kl}(\dot{\xi}_1 - \dot{\xi}_2) + N\frac{I}{j\omega C_0} \\ F_2 = -jZ_0 \tan\frac{kl}{2}\dot{\xi}_2 + \frac{Z_0}{j\sin kl}(\dot{\xi}_1 - \dot{\xi}_2) + N\frac{I}{j\omega C_0} \\ V = \frac{I}{j\omega C_0} + \frac{N}{j\omega C_0}(\dot{\xi}_1 - \dot{\xi}_2) \end{cases} \tag{S4}$$

where Z_0, C_0 and *N* are the acoustic impedance, equivalent capacitance and electro-mechanical conversion coefficient of the piezoelectric transducer, respectively. The 3-port network of the piezoelectric transducer (Fig.3d) is obtained based on equation (S4).

Note.S4. Deduction of the relationship between the reflected pulsed amplitude A_r and the pressure P

The frequency response Z_{ms} of the PMU monitoring system can be described by the developed equation (1), which is a function of frequency ω and the variable resistance R_v. The time-domain function $x(t)$ of the emitted pulse is transformed into a frequency-domain function $X(\omega)$ through a Fourier transform. The response of the PMU monitoring system under $x(t)$ excitation is obtained by superimposing Z_{ms} and $X(\omega)$ (equation (S5)). The $Y(\omega,R_v)$ is the frequency-domain function of the reflected pulse. The time-domain solution $y(t,R_v)$ is then obtained by performing an inverse Fourier transform on $Y(\omega,R_v)$ (equation (S6)). Thus, the reflected pulsed amplitude A_r can be obtained, which is the difference between the maximum and minimum values of $y(t,R_v)$, which is a function of R_v (equation (S7)). The calculation results in Fig.2g demonstrate a linear relationship between A_r and R_v, then $f_1(R_v)$ can be described by equation (S8), where K_1 is a constant value. The experiment results in Fig.3a also demonstrate a linear relationship between the variable resistance R_v and the pressure P, then equation (S9) can be established with a constant value K_2. Thus, the reflected pulsed amplitude A_r can be described as equation (S10), verifying the linear relationship between the pressure P and the amplitude A_r.

$$Y(\omega,R_v)=\mathcal{F}\{x(t)\}\cdot Z_{ms}(\omega,R_v) \tag{S5}$$

$$y(t,R_v)=\mathcal{F}^{-1}\{Y(\omega,R_v)\} \tag{S6}$$

$$A_r=\max(y(t,R_v))-\min(y(t,R_v))=f_1(R_v) \tag{S7}$$

$$f_1(R_v)=K_1\cdot R_v \tag{S8}$$

$$R_v=K_2\cdot P \tag{S9}$$

$$A_r=K_1\cdot K_2\cdot P \tag{S10}$$

Note.S5. The differentiation between reflections from the CUS and substrate interface

The reflected pulse from CUS consists of two parts: the reflection of the CUS surface and the reflection of the substrate interface (corresponding to heart wall interface in *in vivo* experiments) (Fig.S8a). The reflected pulse of the CUS surface carries the monitored pressure signals, which varies with the fluctuation of the pressure (variation part). The reflected pulse of the heart wall is induced by the interface between CUS and the substrate, which is not affected by the pressure and remains constant (constant part). Thus, the reflected pulse can be represented as the superposition of the variation part and constant component, allowing for the differentiation between reflections of the heart wall and the CUS. Then, the reflected pulse amplitude can be divided as follows:

$$A_r = A_{r\text{-}interface} + A_{r\text{-}CUS} = A_0 + K \times p \qquad \text{(S11)}$$

where $A_{r\text{-}interface}$ and $A_{r\text{-}CUS}$ are the reflected pulse amplitudes induced by the heart wall interface and CUS surface, respectively. The A_0 is the constant value of $A_{r\text{-}interface}$. And the K and p are the sensitivity of CUS and the pressure value, respectively.

The mechanism of the PMU principle can provide theoretical support for the differentiation method. Theoretical results of reflected pulse amplitudes are shown in Fig.S8b and Fig.2g, where the reflected pulse amplitude A_r is zero when the resistance R_v is zero. This is because there is no interface influence in the theoretical model. Thus, the substrate interface has no contribution to the reflected pulse amplitude. Experimental results of reflected pulse amplitudes are shown in Fig.S8c and Fig.2h, where the reflected pulse amplitude A_r is a finite value instead of zero when the resistance R_v is zero. This is attributed to the existence of the substrate interface in the experimental setup, which causes the constant part in the reflected pulse amplitude. Therefore, the reflection amplitude of the heart wall interface can be determined through the reflected pulse amplitude with R_v at 0 Ω. Then, the reflected pulse amplitude of CUS ($A_{r\text{-}CUS}$) can be obtained by subtracting the reflected pulse amplitude induced by the heart wall interface ($A_{r\text{-}interface}$).

Note.S6. The influence of biological tissue thickness on the reflected pulse amplitude

Biological tissues have attenuation effects on ultrasound, affecting the ultrasound signals' intensity. Therefore, the attenuation should be considered for the measurement process of the PMU system. Compared to electromagnetic waves, ultrasound has much weaker attenuation in biological tissues (-3 dB/cm versus -1 dB/cm) (*29*), making it a more efficient communication method within the body. When the PMU device is fabricated, the calibration process is carried out to obtain the relationship between the reflected pulse amplitude A_r and pressure value P. After that, the pressure value P can be deduced according to the reflected pulse amplitude A_r obtained by the PMU device. Therefore, there is no need for additional initial calibration for PMU devices during use. In clinical applications, the specific fat thickness of patients can be considered to improve the measurement accuracy of the PMU system. Different fat thicknesses will cause different changes in the reflected pulse amplitudes. The ultrasonic attenuation of fat can be described by the attenuation factor α as follows:

$$\alpha = A/A_0 = \mathrm{e}^{-\beta t} \tag{S12}$$

where t represents a specific fat thickness of patients, and β is the attenuation coefficient of ultrasound in fat. The A and A_0 are amplitudes at the initial position and distance t, respectively. Therefore, the reflected pulse amplitude can be corrected by equation (S12), avoiding the influence caused by different fat thicknesses.

Note.S7. Discussion on the spectrum of reflected pulse when variable resistor R_v is 0 Ω

The experimental results of the spectrum of the PMU system are shown in Fig.S9c. And the spectrum at R_v = 0 Ω has two different peak frequencies of 0.71 MHz (Peak I) and 0.57 MHz (Peak II). This phenomenon can be explained by the theoretical model of the PMU principle. The theoretical results of the spectrum can be derived from equation (1), as shown in Fig.S9d. Results demonstrate that the peak frequency of the reflected pulse shifts to 0.55 MHz at the 0 Ω condition, consistent with the experimental result (0.57 MHz). The peak frequency remains at 0.75 MHz under other resistance conditions, which also agrees with the experimental solution (0.71 MHz). This is because the 0 Ω condition makes the PMU system become a purely capacitive network, which lacks the damping effect of resistance. However, there is no Peak I frequency for 0 Ω condition in theoretical results, which is inconsistent with experimental results. This is due to the Peak I frequency in the experiment being caused by the interface. But there is no interface influence in the theoretical model, which leads to the disappearance of Peak I frequency. Thus, based on results of the theoretical model, we conclude that the waveform difference in the 0 Ω condition is caused by the superposition of CUS surface reflection (Peak II) and interface reflection (Peak I).

Note.S8. The theoretical analysis of the divergence angle of the main lobe.

The acoustic field of the transducer in CUS has a main lobe and many side lobes (Fig.S11). The PMU is achieved through the main lobe, whose intensity should be maximized. The theoretical formula for the directivity of a circular transducer is as follows:

$$D(\theta)=\left|\frac{\int_0^r \rho J_0(k\rho\sin\theta)d\rho}{kr\sin\theta}\right| \tag{S13}$$

where J_0 is the first-order Bessel function, and r is the radius of the transducer. The ρ and θ are length and angle variables in polar coordinates, respectively. The divergence angle of the main lobe is defined as the difference between the first positive zero (θ_+) and the first negative zero (θ_-) of the equation $D(\theta)=0$.

Note.S9. Optimization analysis for the frequency and size of the piezoelectric transducer.

For a circular transducer with a 2.5 mm radius, the DA decreases to 49.70% with frequency increasing from 1 MHz to 2 MHz, but decreases only to 33.10% with frequency increasing from 1 MHz to 3 MHz. Therefore, 2 MHz frequency can enable the transducer to have both high energy intensity of the main lobe and long communication distance. Due to limited internal space inside the human body, the diameter of the transducer should be minimized as much as possible. However, the DA increases sharply as the transducer diameter decreases, severely reducing the energy intensity of the main lobe. Thus, the transducer diameter should be determined to balance both space occupancy and directionality. For a circular transducer with 2 MHz frequency, the divergence angle increases by only 20% with the radius decreasing from 3 mm to 2.5 mm, but increases by 50.31% with the radius decreasing from 3 mm to 2 mm. Therefore, 2.5 mm radius can enable the transducer to have good directivity and a small device size.

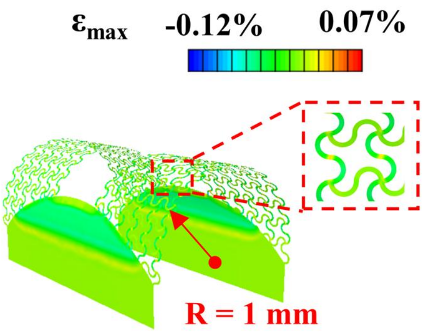

Fig.S1. Simulation results of maximum strain distribution of the stretchable electrode layer during bending deformation.

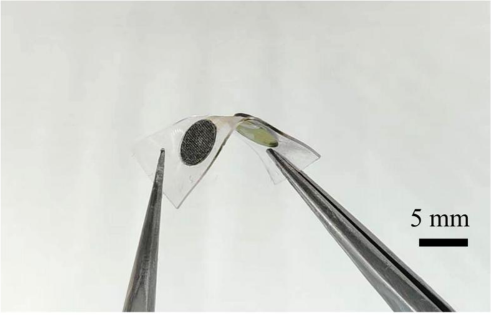

Fig.S2. Optical diagram of the CUS under bending configuration.

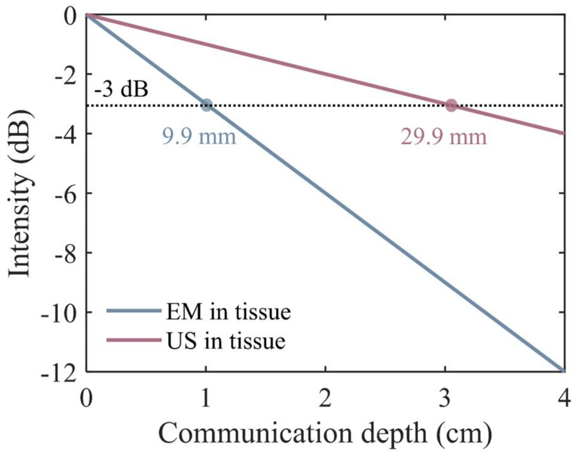

Fig.S3. The energy attenuation with propagation distance *in vivo* for both electromagnetic waves and ultrasounds.

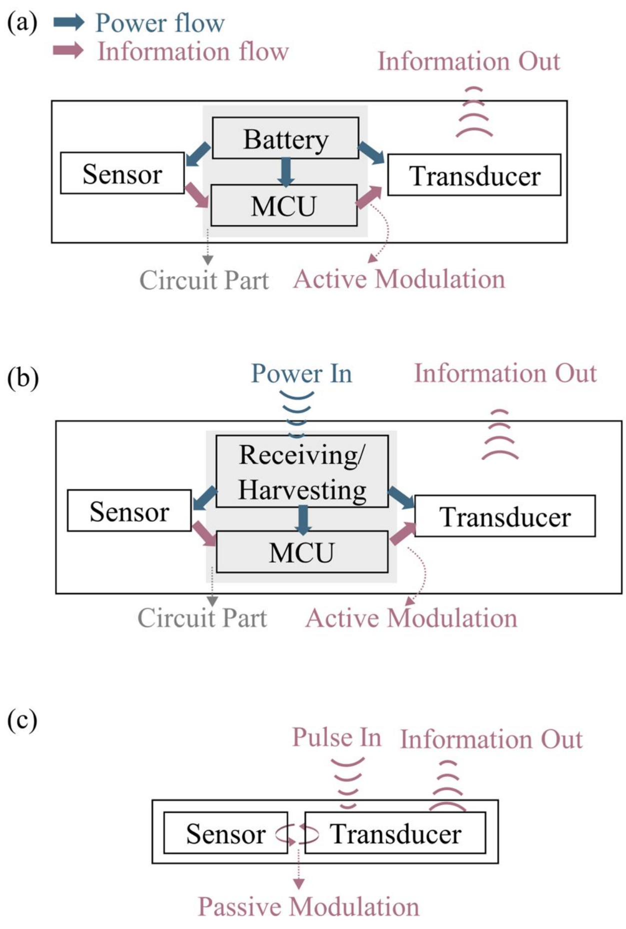

Fig.S4. The block diagrams of current implantable biomedical devices and PMU devices. (a) Current devices with both active power supply and active communication. (b) Current devices with passive power supply but active communication. (c) PMU devices with both passive power supply and passive communication.

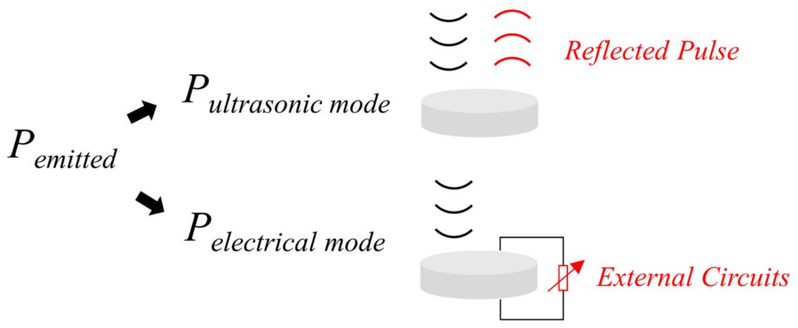

Fig.S5. The energy distribution in PMU. The energy distribution includes energy reflected backward as ultrasound pulses (ultrasonic mode) and energy depleted on the external circuit (electrical mode).

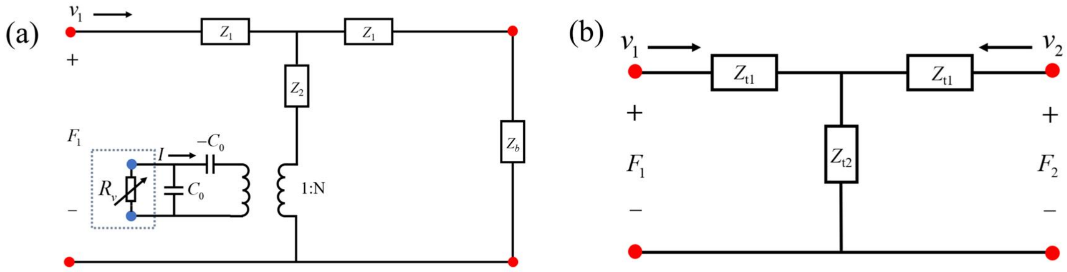

Fig.S6. Network models of the PMU system and the human tissue layer. (a) A variable resistor R_v is connected in series at the electrical port of the network of the piezoelectric transducer. (b) The network model of the human tissue layer.

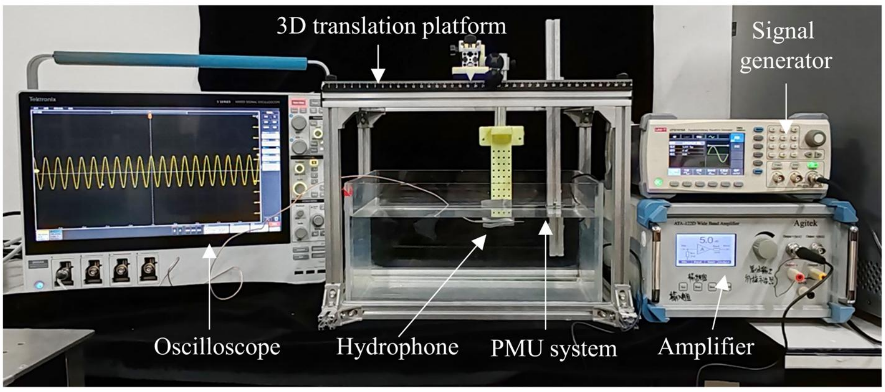

Fig.S7. The *in vitro* experiment setup for analysis of the PMU method. The system includes a hydrophone, a PMU system, an oscilloscope, a customized 3D translation platform, a signal generator and an amplifier.

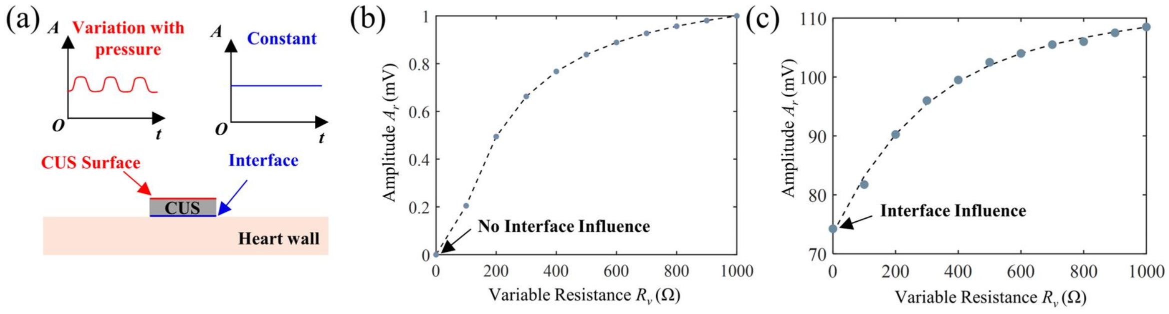

Fig.S8. The differentiation of reflections from the CUS and the heart wall. (a) The schematic of the varying reflection caused by CUS and constant reflection caused by the heart wall interface, respectively. (b) Theoretical results of reflected pulse amplitudes under different resistance R_v without interface influence. (c) Experimental results of reflected pulse amplitudes under different resistance R_v with interface influence.

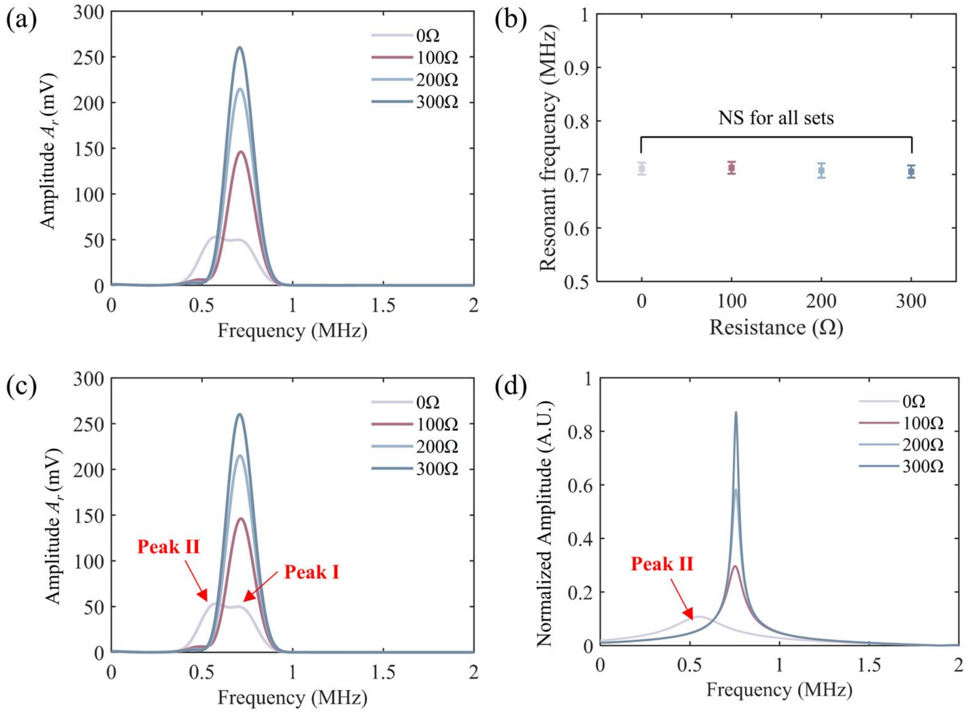

Fig.S9. Spectral analysis of waveforms obtained through the PMU method. (a) The frequency distribution of all reflected pulses with a resonant frequency of around 0.75 MHz. (b) Comparison of the peak frequency of reflected pulses under different resistance R_v. (c,d) Peak frequencies of (c) experimental results and (d) theoretical results about the frequency response of the PMU system.

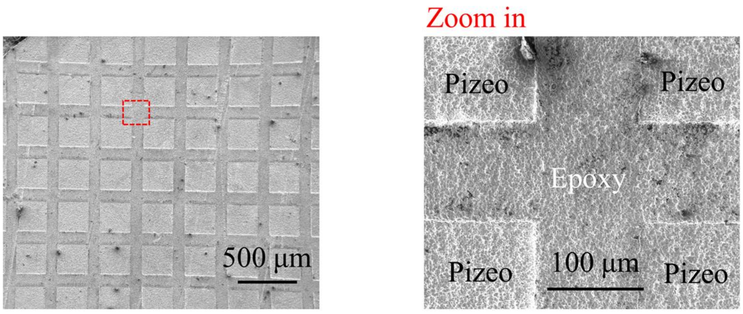

Fig.S10. The scanning electron microscope (SEM) diagram of the 1-3 composite piezoelectric material. This material includes multiple $Pb(Zr,Ti)O_3$ (PZT) microcolumns, with epoxy filled between them. In the thickness vibration mode, the 1-3 composite has a high piezoelectric coefficient d_{33}, which is suitable for amplitude modulation in CUS. The composite has better acoustic impedance matching performance with human tissue than bulk PZT.

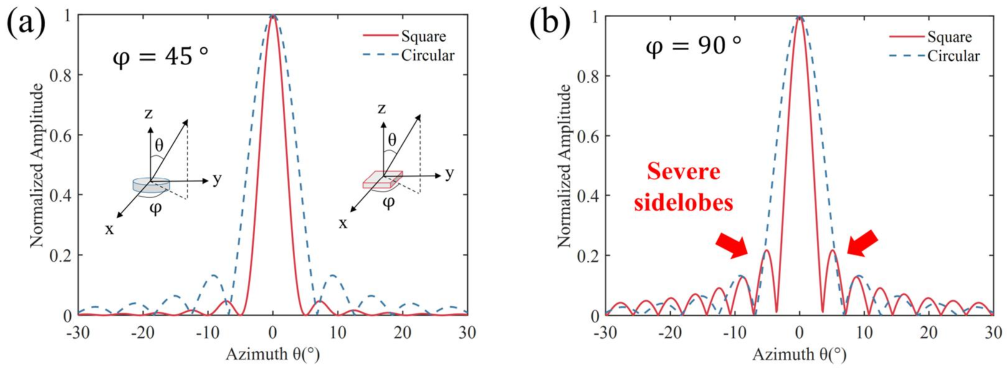

Fig.S11. Comparison of acoustic field directionality between a circular transducer and a square transducer. (a) The directionality of the square transducer is not uniform in different directions, which affects the uniformity and sensitivity of the PMU. (b) The circular transducer has the same directionality at all directions, which has stable communication performance.

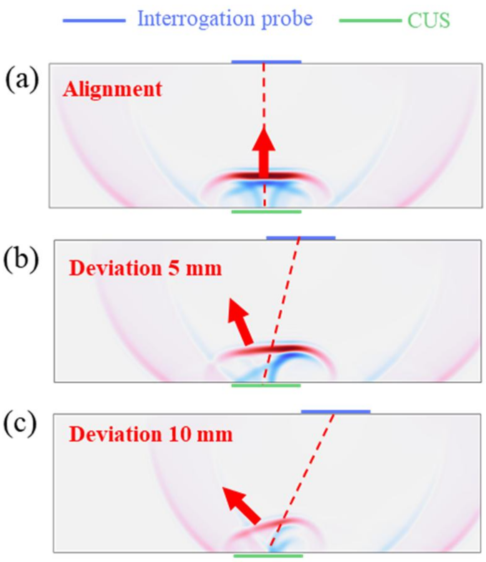

Fig.S12. Simulated results of the acoustic field when the interrogation probe is offset from the CUS at different distances. (a) The reflected pulse returns directly toward the interrogation probe when it is aligned with the CUS. (b,c) The direction of the reflected pulse deviates when the interrogation probe is offset from the CUS.

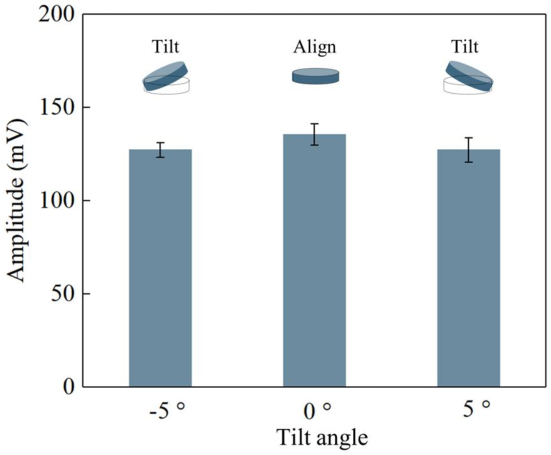

Fig.S13. The reflected pulse amplitudes at different tilt angles under a fixed emitted pulse amplitude.

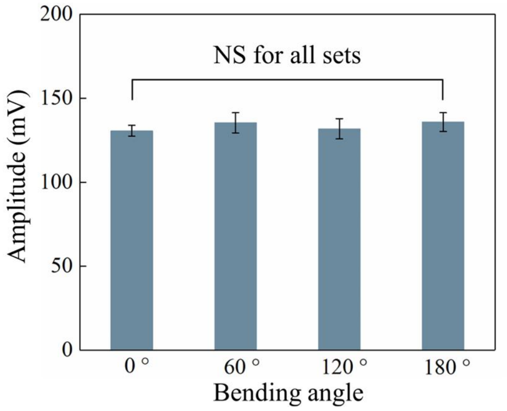

Fig.S14. The reflected pulse amplitudes at different bending angles under a fixed emitted pulse amplitude.

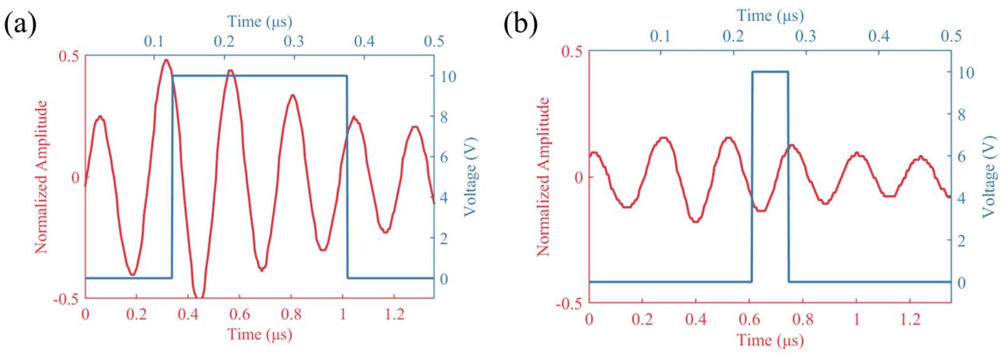

Fig.S15. Comparison of reflected ultrasound pulses corresponding to different time widths of emitted ultrasound pulses. (a) The reflected pulse corresponding to long time width (250 ns) of the emitted pulse. (b) The reflected pulse corresponding to short time width (50 ns) of the emitted pulse.

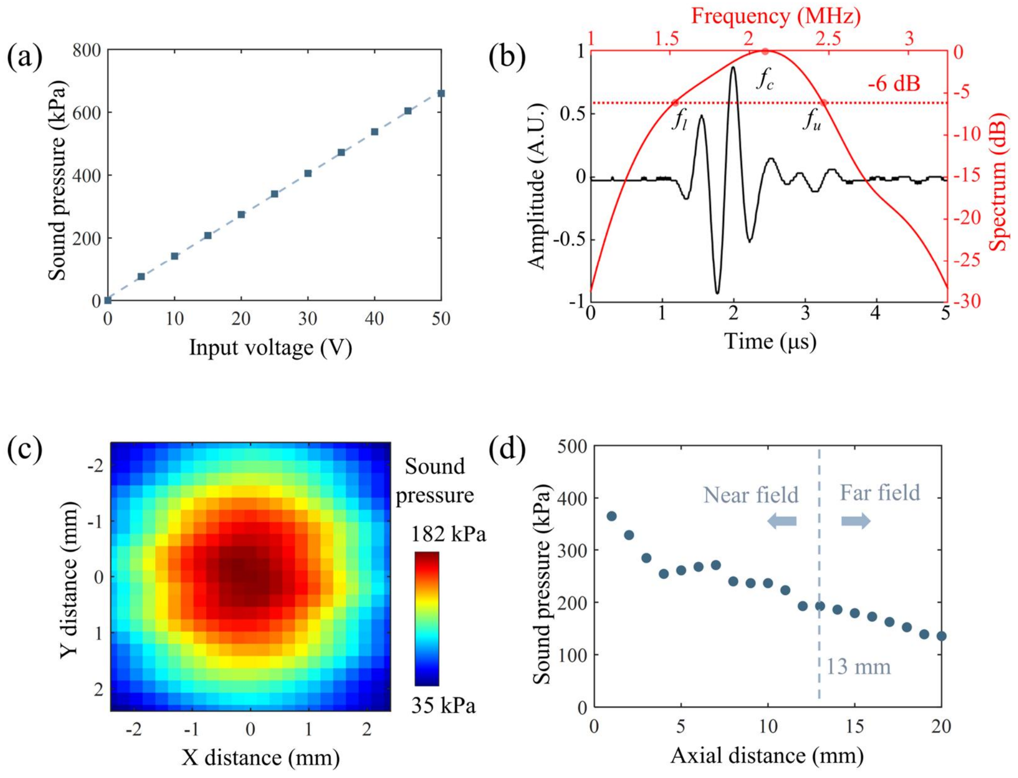

Fig.S16. The characterization results of the interrogation probe. (a) The sound pressure generated by the interrogation probe under different excitation voltages. (b) The pulse-echo response (black) and frequency spectrum (red) of the interrogation probe. (c,d) The cross-sectional (c) and axial (d) distribution of sound pressure intensity emitted by the interrogation probe.

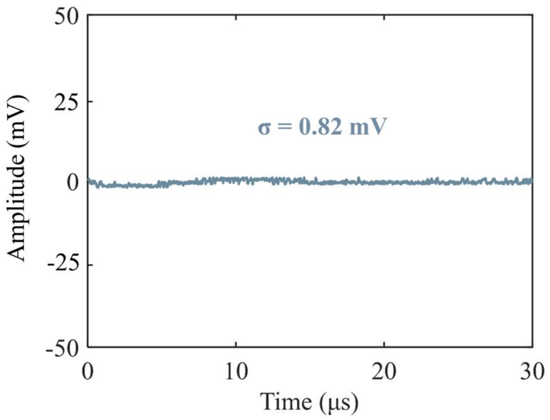

Fig.S17. The output signals of the ultrasonic control equipment in a resting state.

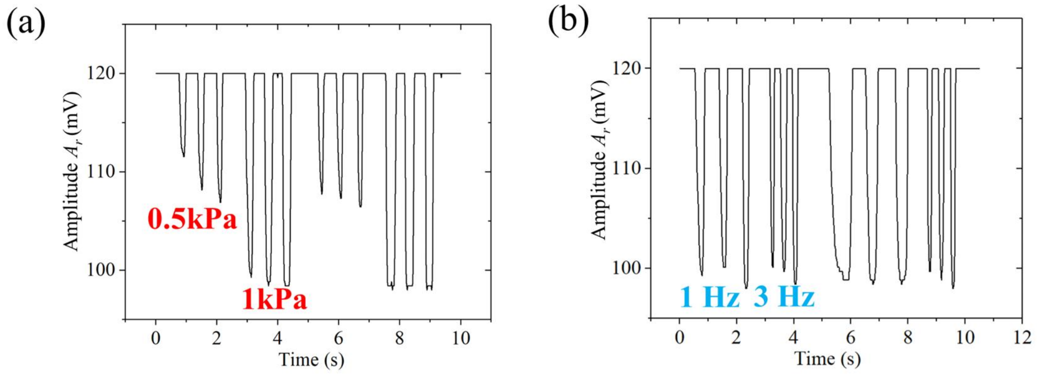

Fig.S18. Sensing performance of the CUS under (a) different loading pressures and (b) different loading frequencies.

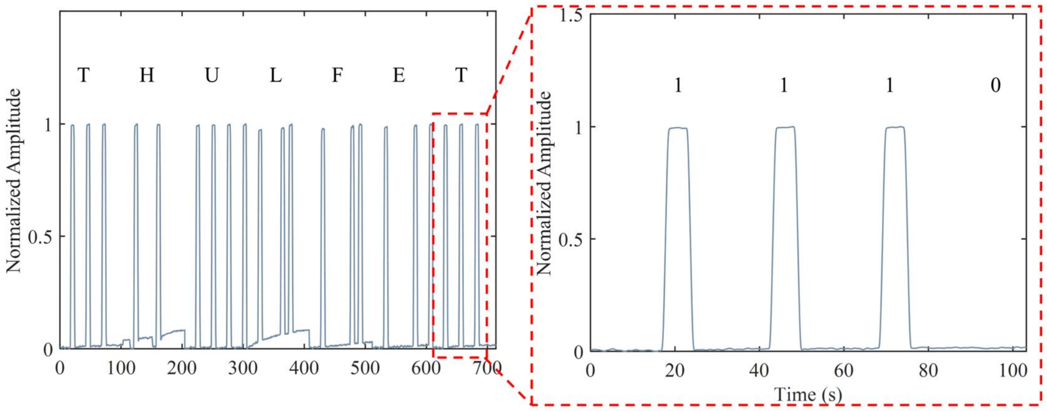

Fig.S19. The binary encoding communication of CUS based on ASCII encoding protocol.

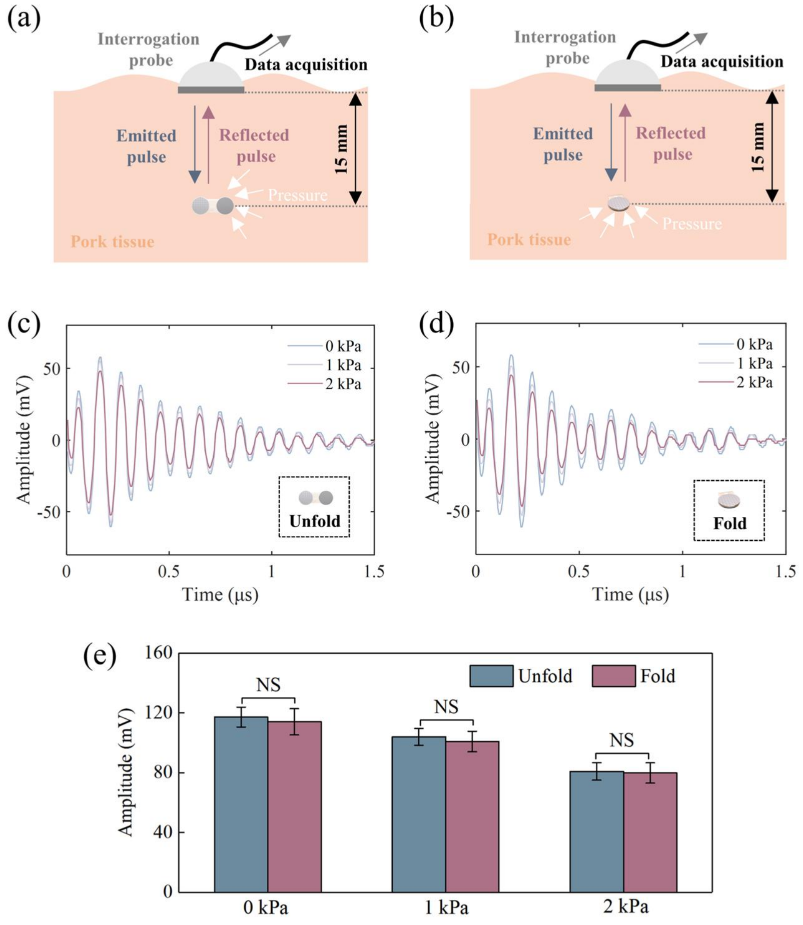

Fig.S20. The characterization of pressure monitoring performance of the CUS in folded and unfolded states. (a,b) The schematic of CUS in unfolded and folded state, respectively. (c,d) The pressure monitoring results of CUS in unfolded and folded state, respectively. (e) Comparison of pressure monitoring results between the device in folded and unfolded states.

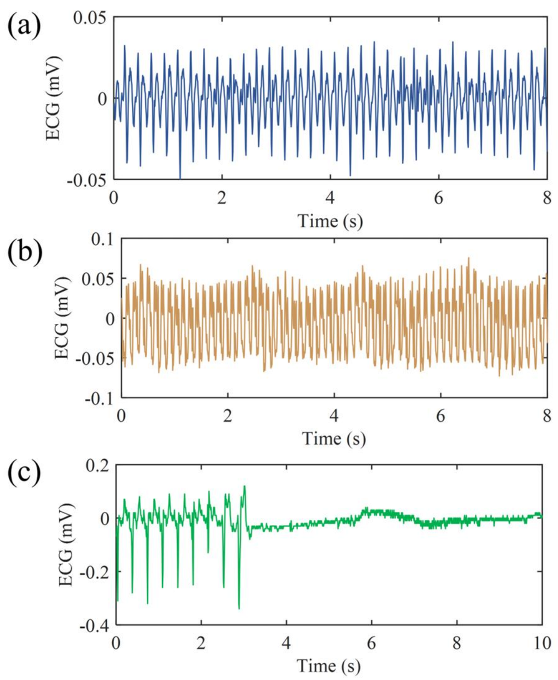

Fig.S21. Rabbit's (a) normal ECG, (b) ECG after injecting dopamine to induce heart failure, and (c) ECG when injecting KCL solution to induce cardiac arrest.

REFERENCES AND NOTES

1. J. Liu, N. Liu, Y. Xu, M. Wu, H. Zhang, Y. Wang, Y. Yan, A. Hill, R. Song, Z. Xu, M. Park, Y. Wu, J. L. Ciatti, J. Gu, H. Luan, Y. Zhang, T. Yang, H.-Y. Ahn, S. Li, W. Z. Ray, C. K. Franz, M. R. M. Ewan, Y. Huang, C. W. Hammill, H. Wang, J. A. Rogers, Bioresorbable shape-adaptive structures for ultrasonic monitoring of deep-tissue homeostasis. *Science* **383**, 1096–1103 (2024).

2. Y. Zhang, N. Zheng, Y. Cao, F. Wang, P. Wang, Y. Ma, B. Lu, G. Hou, Z. Fang, Z. Liang, M. Yue, Y. Li, Y. Chen, J. Fu, J. Wu, T. Xie, X. Feng, Climbing-inspired twining electrodes using shape memory for peripheral nerve stimulation and recording. *Sci. Adv.* **5**, eaaw1066 (2019).

3. S. Li, D. Lu, S. Li, J. Liu, Y. Xu, Y. Yan, J. Z. Rodriguez, H. Bai, R. Avila, S. Kang, X. Ni, H. Luan, H. Guo, W. Bai, C. Wu, X. Zhou, Z. Hu, M. A. Pet, C. W. Hammill, M. R. Macewan, W. Z. Ray, Y. Huang, J. A. Rogers, Bioresorbable, wireless, passive sensors for continuous pH measurements and early detection of gastric leakage. *Sci. Adv.* **10**, eadj0268 (2024).

4. J. Lee, V. Leung, A.-H. Lee, J. Huang, P. Asbeck, P. P. Mercier, S. Shellhammer, L. Larson, F. Laiwalla, A. Nurmikko, Neural recording and stimulation using wireless networks of microimplants. *Nat. Electron.* **4**, 604–614 (2021).

5. J. Kim, M. I. Bury, K. Kwon, J. Y. Yoo, N. V. Halstead, H.-S. Shin, S. Li, S. M. Won, M.-H. Seo, Y. Wu, D. Y. Park, M. Kini, J. W. Kwak, S. R. Madhvapathy, J. L. Ciatti, J. H. Lee, S. Kim, H. Ryu, K. Yamagishi, H.-J. Yoon, S. S. Kwak, B. Kim, Y. Huang, L. C. Halliday, E. Y. Cheng, G. A. Ameer, A. K. Sharma, J. A. Rogers, A wireless, implantable bioelectronic system for monitoring urinary bladder function following surgical recovery. *Proc. Natl. Acad. Sci. U.S.A.* **121**, e2400868121 (2024).

6. D. Jiang, B. Shi, H. Ouyang, Y. Fan, Z. L. Wang, Z. Li, Emerging implantable energy harvesters and self-powered implantable medical electronics. *ACS Nano* **14**, 6436–6448 (2020).

7. J. H. Koo, J.-K. Song, D.-H. Kim, D. Son, Soft implantable bioelectronics. *ACS Mater. Lett.* **3**, 1528–1540 (2021).

8. D. Seo, R. M. Neely, K. Shen, U. Singhal, E. Alon, J. M. Rabaey, J. M. Carmena, M. M. Maharbiz, Wireless recording in the peripheral nervous system with ultrasonic neural dust. *Neuron* **91**, 529–539 (2016).

9. S. Yoo, J. Lee, H. Joo, S.-H. Sunwoo, S. Kim, D.-H. Kim, Wireless power transfer and telemetry for implantable bioelectronics. *Adv. Healthc. Mater.* **10**, e2100614 (2021).

10. H. Wang, X. Yu, Wireless powering solution for implantable electronics based on ultra-low frequency magnetic energy focusing. *Natl. Sci. Rev.* **11**, nwae140 (2024).

11. J. Kim, A. Banks, Z. Xie, S. Y. Heo, P. Gutruf, J. W. Lee, S. Xu, K.-I. Jang, F. Liu, G. Brown, J. Choi, J. H. Kim, X. Feng, Y. Huang, U. Paik, J. A. Rogers, Miniaturized flexible electronic systems with wireless power and near-field communication capabilities. *Adv. Funct. Mater.* **25**, 4761–4767 (2015).

12. B. Gil, S. Anastasova, G.-Z. Yang, Low-powered implantable devices activated by ultrasonic energy transfer for physiological monitoring in soft tissue via functionalized electrochemical electrodes. *Biosens. Bioelectron.* **182**, 113175 (2021).

13. R. Hinchet, H.-J. Yoon, H. Ryu, M.-K. Kim, E.-K. Choi, D.-S. Kim, S.-W. Kim, Transcutaneous ultrasound energy harvesting using capacitive triboelectric technology. *Science* **365**, 491–494 (2019).

14. V. Nair, A. N. Dalrymple, Z. Yu, G. Balakrishnan, C. J. Bettinger, D. J. Weber, K. Yang, J. T. Robinson, Miniature battery-free bioelectronics. *Science* **382**, eabn4732 (2023).

15. C. Yu, M. Shi, S. He, M. Yao, H. Sun, Z. Yue, Y. Qiu, B. Liu, L. Liang, Z. Zhao, F. Yao, H. Zhang, J. Li, Chronological adhesive cardiac patch for synchronous mechanophysiological monitoring and electrocoupling therapy. *Nat. Commun.* **14**, 6226 (2023).

16. Z. Xie, R. Avila, Y. Huang, J. A. Rogers, Flexible and stretchable antennas for biointegrated electronics. *Adv. Mater.* **32**, e1902767 (2020).

17. W. Lyu, Y. Ma, S. Chen, H. Li, P. Wang, Y. Chen, X. Feng, Flexible ultrasonic patch for accelerating chronic wound healing. *Adv. Healthc. Mater.* **10**, 2100785 (2021).

18. M. Lin, H. Hu, S. Zhou, S. Xu, Soft wearable devices for deep-tissue sensing. *Nat. Rev. Mater.* **7**, 850–869 (2022).

19. M. Song, P. Jayathurathnage, E. Zanganeh, M. Krasikova, P. Smirnov, P. Belov, P. Kapitanova, C. Simovski, S. Tretyakov, A. Krasnok, Wireless power transfer based on novel physical concepts. *Nat. Electron.* **4**, 707–716 (2021).

20. F. Wang, P. Jin, Y. Feng, J. Fu, P. Wang, X. Liu, Y. Zhang, Y. Ma, Y. Yang, A. Yang, X. Feng, Flexible Doppler ultrasound device for the monitoring of blood flow velocity. *Sci. Adv.* **7**, eabi9283 (2021).

21. J. Charthad, M. J. Weber, T. C. Chang, A. Arbabian, A mm-sized implantable medical device (IMD) with ultrasonic power transfer and a hybrid bi-directional data link. *IEEE J. Solid State Circuits* **50**, 1741–1753 (2015).

22. D. Seo, J. M. Carmena, J. M. Rabaey, M. M. Maharbiz, E. Alon, Model validation of untethered, ultrasonic neural dust motes for cortical recording. *J. Neurosci. Meth.* **244**, 114–122 (2015).

23. P. Jin, J. Fu, F. Wang, Y. Zhang, P. Wang, X. Liu, Y. Jiao, H. Li, Y. Chen, Y. Ma, X. Feng, A flexible, stretchable system for simultaneous acoustic energy transfer and communication. *Sci. Adv.* **7**, eabg2507 (2021).

24. D. Li, J. Zhou, Z. Zhao, X. Huang, H. Li, Q. Qu, C. Zhou, K. Yao, Y. Liu, M. Wu, J. Su, R. Shi, Y. Huang, J. Wang, Z. Zhang, Y. Liu, Z. Gao, W. Park, H. Jia, X. Guo, J. Zhang, P. Chirarattananon, L. Chang, Z. Xie, X. Yu, Battery-free, wireless, and electricity-driven soft swimmer for water quality and virus monitoring. *Sci. Adv.* **10**, eadk6301 (2024).

25. H. Hu, H. Huang, M. Li, X. Gao, L. Yin, R. Qi, R. S. Wu, X. Chen, Y. Ma, K. Shi, C. Li, T. M. Maus, B. Huang, C. Lu, M. Lin, S. Zhou, Z. Lou, Y. Gu, Y. Chen, Y. Lei, X. Wang, R. Wang, W. Yue, X. Yang, Y. Bian, J. Mu, G. Park, S. Xiang, S. Cai, P. W. Corey, J. Wang, S. Xu, A wearable cardiac ultrasound imager. *Nature* **613**, 667–675 (2023).

26. H. Li, Y. Ma, Z. Liang, Z. Wang, Y. Cao, Y. Xu, H. Zhou, B. Lu, Y. Chen, Z. Han, S. Cai, X. Feng, Wearable skin-like optoelectronic systems with suppression of motion artifacts for cuff-less continuous blood pressure monitor. *Natl. Sci. Rev.* **7**, 849–862 (2020).

27. J. Jhang, S. Park, S. J. Liu, D. D. O'Keefe, S. Han, A top-down slow breathing circuit that alleviates negative affect in mice. *Nat. Neurosci.* **27**, 2455–2465 (2024).

28. E. Akoumianaki, S. M. Maggiore, F. Valenza, G. Bellani, A. Jubran, S. H. Loring, P. Pelosi, D. Talmor, S. Grasso, D. Chiumello, C. Guérin, N. Patroniti, V. M. Ranieri, L. Gattinoni, S. Nava, P.-P. Terragni, A. Pesenti, M. Tobin, J. Mancebo, L. Brochard, PLUG Working Group (Acute Respiratory Failure Section of the European Society of Intensive Care Medicine), The application of esophageal pressure measurement in patients with respiratory failure. *Am. J. Respir. Crit. Care Med.* **189**, 520–531 (2014).

29. S. Sonmezoglu, J. R. Fineman, E. Maltepe, M. M. Maharbiz, Monitoring deep-tissue oxygenation with a millimeter-scale ultrasonic implant. *Nat. Biotechnol.* **39**, 855–864 (2021).

30. M. Kiani, M. Ghovanloo, A 13.56-Mbps pulse delay modulation based transceiver for simultaneous near-field data and power transmission. *IEEE Trans. Biomed. Circuits Syst.* **9**, 1–11 (2014).

31. R. P. Khokle, F. Franco, S. C. de Freitas, K. P. Esselle, M. C. Heimlich, D. J. Bokor, Eddy current–tunneling magneto-resistive sensor for micromotion detection of a tibial orthopaedic implant. *IEEE Sens. J.* **19**, 1285–1292 (2018).

32. B. C. Johnson, K. Shen, D. Piech, M. M. Ghanbari, K. Y. Li, R. Neely, J. M. Carmena, M. M. Maharbiz, R. Muller, paper presented at the *2018 IEEE Custom Integrated Circuits Conference (CICC)*, San Diego, CA, 8 to 11 April 2018.

33. L. Maini, V. Genovés, R. Furrer, N. Cesarovic, C. Hierold, C. Roman, An in vitro demonstration of a passive, acoustic metamaterial as a temperature sensor with mK resolution for implantable applications. *Microsyst. Nanoeng.* **10**, 8 (2024).

34. C. M. Boutry, L. Beker, Y. Kaizawa, C. Vassos, H. Tran, A. C. Hinckley, R. Pfattner, S. Niu, J. Li, J. Claverie, Z. Wang, J. Chang, P. M. Fox, Z. Bao, Biodegradable and flexible arterial-pulse sensor for the wireless monitoring of blood flow. *Nat. Biomed. Eng.* **3**, 47–57 (2019).

35. F. Stauffer, Q. Zhang, K. Tybrandt, B. L. Zambrano, J. Hengsteler, A. Stoll, C. Trüeb, M. Hagander, J.-M. Sujata, F. Hoffmann, J. S. Stekhoven, J. Quack, H. Zilly, J. Goedejohann, M. P. Schneider, T. M. Kessler, W. R. Taylor, R. Küng, J. Vörös, Soft electronic strain sensor with chipless wireless readout: Toward real-time monitoring of bladder volume. *Adv. Mater. Technol.* **3**, 1800031 (2018).

36. V. M. Bogomol'nyi, Electroelasticity relations and fracture mechanics of piezoelectric structures. *Appl. Mech. Rev.* **60**, 21–36 (2007).

37. C. Hellmich, N. Ukaj, B. Smeets, H. van Oosterwyck, N. Filipovic, L. Zelaya-Lainez, J. Kalliauer, S. Scheiner, Hierarchical biomechanics: concepts, bone as prominent example, and perspectives beyond. *Appl. Mech. Rev.* **74**, 030802 (2022).

38. L. Jiang, Y. Yang, R. Chen, G. Lu, R. Li, D. Li, M. S. Humayun, K. K. Shung, J. Zhu, Y. Chen, Q. Zhou, Flexible piezoelectric ultrasonic energy harvester array for bio-implantable wireless generator. *Nano Energy* **56**, 216–224 (2019).

39. Z. Zhan, R. Lin, V.-T. Tran, J. An, Y. Wei, H. Du, T. Tran, W. Lu, Paper/carbon nanotube-based wearable pressure sensor for physiological signal acquisition and soft robotic skin. *ACS Appl. Mater. Interfaces* **9**, 37921–37928 (2017).

40. S. Chen, J. Qi, S. Fan, Z. Qiao, J. C. Yeo, C. T. Lim, Flexible wearable sensors for cardiovascular health monitoring. *Adv. Healthc. Mater.* **10**, 2100116 (2021).

41. L. Testai, A. Martelli, A. A. Marino, V. D'Antongiovanni, F. Ciregia, L. Giusti, A. Lucacchini, S. Chericoni, M. C. Breschi, V. Calderone, The activation of mitochondrial BK potassium channels contributes to the protective effects of naringenin against myocardial ischemia/reperfusion injury. *Biochem. Pharmacol.* **85**, 1634–1643 (2013).

42. J. A. I. Virag, R. M. Lust, Coronary artery ligation and intramyocardial injection in a murine model of infarction. *J. Vis. Exp.* doi: 10.3791/2581, 2581 (2011).

43. C.-H. Chiang, S. M. Won, A. L. Orsborn, K. J. Yu, M. Trumpis, B. Bent, C. Wang, Y. Xue, S. Min, V. Woods, C. Yu, B. H. Kim, S. B. Kim, R. Huq, J. Li, K. J. Seo, F. Vitale, A. Richardson, H. Fang, Y. Huang, K. Shepard, B. Pesaran, J. A. Rogers, J. Viventi, Development of a neural interface for high-definition, long-term recording in rodents and nonhuman primates. *Sci. Transl. Med.* **12**, eaay4682 (2020).

44. J. D. Schmitto, S. A. Mokashi, L. S. Lee, A. F. Popov, K. O. Coskun, S. Sossalla, C. Sohns, R. M. Bolman III, L. H. Cohn, F. Y. Chen, Large animal models of chronic heart failure (CHF). *J. Surg. Res.* **166**, 131–137 (2011).

45. C. Wang, X. Li, H. Hu, L. Zhang, Z. Huang, M. Lin, Z. Zhang, Z. Yin, B. Huang, H. Gong, S. Bhaskaran, Y. Gu, M. Makihata, Y. Guo, Y. Lei, Y. Chen, C. Wang, Y. Li, T. Zhang, Z. Chen, A. P. Pisano, L. Zhang, Q. Zhou, S. Xu, Monitoring of the central blood pressure waveform via a conformal ultrasonic device. *Nat. Biomed. Eng.* **2**, 687–695 (2018).

46. J. Wan, Z. Y. Nie, J. Xu, Z. X. Zhang, S. L. Yao, Z. H. Xiang, X. Lin, Y. X. Lu, C. Xu, P. C. Zhao, Y. R. Wang, J. Y. Zhang, Y. Z. Wang, S. T. Zhang, J. Z. Wang, W. T. Man, M. Zhang, M. D. Han, Millimeter-scale magnetic implants paired with a fully integrated wearable device for wireless biophysical and biochemical sensing. *Sci. Adv.* **10**, eadm9314 (2024).

47. K. Kwon, J. U. Kim, S. M. Won, J. Z. Zhao, R. Avila, H. L. Wang, K. S. Chun, H. Jang, K. H. Lee, J. H. Kim, S. Yoo, Y. J. Kang, J. Kim, J. Lim, Y. Park, W. Lu, T. I. Kim, A. Banks, Y. G. Huang, J. A. Rogers, A battery-less wireless implant for the continuous monitoring of vascular pressure, flow rate and temperature. *Nat. Biomed. Eng.* **7**, 1215–1228 (2023).

48. Y. S. Choi, R. T. Yin, A. Pfenniger, J. Koo, R. Avila, K. Benjamin Lee, S. W. Chen, G. Lee, G. Li, Y. Qiao, A. Murillo-Berlioz, A. Kiss, S. Han, S. M. Lee, C. Li, Z. Xie, Y.-Y. Chen, A. Burrell, B. Geist, H. Jeong, J. Kim, H.-J. Yoon, A. Banks, S.-K. Kang, Z. J. Zhang, C. R. Haney, A. V. Sahakian, D. Johnson, T. Efimova, Y. Huang, G. D. Trachiotis, B. P. Knight, R. K. Arora, I. R. Efimov, J. A. Rogers, Fully implantable and bioresorbable cardiac pacemakers without leads or batteries. *Nat. Biotechnol.* **39**, 1228–1238 (2021).

49. S.-Y. Yang, V. Sencadas, S. S. You, N. Z.-X. Jia, S. S. Srinivasan, H.-W. Huang, A. E. Ahmed, J. Y. Liang, G. Traverso, Powering implantable and ingestible electronics. *Adv. Funct. Mater.* **31**, 2009289 (2021).

50. D. C. Bock, A. C. Marschilok, K. J. Takeuchi, E. S. Takeuchi, Batteries used to power implantable biomedical devices. *Electrochim. Acta* **84**, 155–164 (2012).

51. Z. Gao, Y. Zhou, J. Zhang, J. Foroughi, S. Peng, R. H. Baughman, Z. L. Wang, C. H. Wang, Advanced energy harvesters and energy storage for powering wearable and implantable medical devices. *Adv. Mater.* **36**, e2404492 (2024).

52. Y. Song, J. Min, Y. Yu, H. Wang, Y. Yang, H. Zhang, W. Gao, Wireless battery-free wearable sweat sensor powered by human motion. *Sci. Adv.* **6**, eaay9842 (2020).
